# Pitfalls of using the risk ratio in meta‐analysis

**DOI:** 10.1002/jrsm.1347

**Published:** 2019-04-11

**Authors:** Ilyas Bakbergenuly, David C. Hoaglin, Elena Kulinskaya

**Affiliations:** ^1^ School of Computing Sciences University of East Anglia Norwich United Kingdom; ^2^ University of Massachusetts Medical School Worcester Massachusetts

**Keywords:** beta‐binomial model, log‐binomial model, relative risk, response ratio, risk difference

## Abstract

For meta‐analysis of studies that report outcomes as binomial proportions, the most popular measure of effect is the odds ratio (OR), usually analyzed as log(OR). Many meta‐analyses use the risk ratio (RR) and its logarithm because of its simpler interpretation. Although log(OR) and log(RR) are both unbounded, use of log(RR) must ensure that estimates are compatible with study‐level event rates in the interval (0, 1). These complications pose a particular challenge for random‐effects models, both in applications and in generating data for simulations. As background, we review the conventional random‐effects model and then binomial generalized linear mixed models (GLMMs) with the logit link function, which do not have these complications. We then focus on log‐binomial models and explore implications of using them; theoretical calculations and simulation show evidence of biases. The main competitors to the binomial GLMMs use the beta‐binomial (BB) distribution, either in BB regression or by maximizing a BB likelihood; a simulation produces mixed results. Two examples and an examination of Cochrane meta‐analyses that used RR suggest bias in the results from the conventional inverse‐variance–weighted approach. Finally, we comment on other measures of effect that have range restrictions, including risk difference, and outline further research.

## 1 INTRODUCTION

For meta‐analysis of studies that report binary outcomes (usually summarized as the number of subjects who had an event and the number who had no event, in a treatment group and a control group), the most popular measure of effect is the odds ratio (OR), usually analyzed in the log scale, as the difference in log‐odds between the two groups. Many meta‐analyses, however, use the risk ratio (RR), the ratio of the probability of an event in the treatment group (π_T_) to that in the control group (π_C_). Importantly, the benefits of analyzing log(RR) are offset by the restrictions π_C_ < 1 and π_T_ < 1, which need to be explicitly applied to their estimates, unlike in the analysis of log(OR). Thus, one must balance the mathematical convenience of the OR against the simpler interpretation of the RR.

When π_C_ and π_T_ are small (eg, <0.1), OR≈RR. If π_C_ or π_T_ is not small, however, RR (also called the relative risk) is often considered a better measure of effect than OR, despite the latter's mathematical convenience. In applications, the RR and its complement, the percentage reduction in risk, have a direct interpretation. Fleiss et al^1^ point out that Cornfield^2^ proposed the OR, in 1951, only because it provided a good approximation to the relative risk (interestingly, Cornfield^2^ did not use the term odds ratio). In general, when π_T_ < π_C_, OR < RR < 1; and when π_T_ > π_C_, OR > RR > 1.^3^ That is, OR is always farther from 1 than RR. Various authors have discussed reasons for choosing RR instead of OR and the ease with which OR can be misinterpreted (eg, Sinclair and Bracken,^4^ Sackett et al.,^5^ Altman et al.,^6^ Deeks,^7^ and Newcombe^8^). The OR is necessary in case‐control studies (where the RR cannot be estimated directly), and it readily allows adjustment for covariates via logistic regression, but those applications are usually separate from meta‐analysis.

When a population consists of strata, it may be possible to summarize an effect more simply if the measure for the entire sample adequately represents the stratum‐specific measures, that is, if the measure of effect can be collapsed over the strata. The RR for the entire sample must lie within the range of the stratum‐specific RRs, but the OR for the entire sample can be closer to 1 than the OR for any of the strata.^9^ Even in ideal cases in which the RR or OR is the same in all strata, however, the corresponding measure for the entire population may not equal that common value. Certain conditions must hold for collapsibility of the RR or the OR.^10^

Methods for meta‐analysis of RRs have received much less attention than methods for ORs, in part because analysis of their performance involves complications arising mainly from the restrictions on the ranges of ${\hat{\pi }}_{C}$ and ${\hat{\pi }}_{T}$. The impact of those complications on actual meta‐analyses is not widely understood and may not be apparent to users. We discuss the role of the restrictions in models for fixed‐effect and, especially, random‐effects meta‐analysis, examine their impact on generation of data for simulation studies and on the results, and deduce their likely contribution to bias in examples and in a sizable number of Cochrane reviews. As background, Section 2 reviews the conventional random‐effects model (REM), which uses the sample log‐odds‐ratio or log‐risk‐ratio as the measure of effect.

To avoid the assumptions and approximations in the conventional methods, an alternative approach bases the analysis on the likelihood for pairs of independent binomial distributions. Section 3 discusses this approach, an application of generalized linear mixed models (GLMMs) with the logit and log transformations as the usual link functions and normal as the usual distribution of random effects. In REMs for the log‐risk‐ratio, the constraint on π_T_ imposes a truncation on the distribution of the random effects; we use simulation to explore the impact on estimation of the between‐study variance (τ ^2^) and the overall log‐risk‐ratio.

The main alternative to the binomial GLMMs is beta‐binomial (BB) regression. The BB distribution arises as a mixture of binomial distributions in which the probability of an event, p, follows a beta distribution. Section 4 reviews the BB distribution and BB regression and discusses its application in meta‐analysis of log‐risk‐ratios. In Section 5, we analyze two examples to compare conventional procedures and the procedures based on BB distributions. Using a collection of 1286 meta‐analyses of RR, in a 2004 Cochrane Library issue, we explore (in Section 6) several practical implications of the restriction on the range of the binomial rates (represented by truncation of the distribution of random effects). Finally, the discussion in Section 7 puts our investigation and results in perspective. We have focused on the RR, but other measures of effect also have range restrictions, including risk difference, response ratio (ie, the log of the ratio of means), and $\arcsin(\sqrt{p})$ for binomial proportions; methods for these need further research.

## 2 CONVENTIONAL REM 

Random‐effects meta‐analysis aims to estimate an overall effect, θ, defined as the mean of a distribution of study‐level effects whose variance is τ ^2^. When τ ^2^ = 0, the REM reduces to the fixed‐effect model. For the usual choice of a normal distribution, the effects in the individual studies are θ_j_∼N(θ,τ ^2^), j = 1,…,K. Study j yields the estimate y_j_ of θ_j_, along with an estimate, $s_{j}^{2}$, of its within‐study variance, $\sigma_{j}^{2}$. For some measures of effect, such as mean difference (ie, the difference between the mean outcome in the treatment group and the mean outcome in the control group), y_j_ and θ_j_ are in the same scale as the data. For other measures, y_j_ comes from applying a transformation to the data of study  j or to a summary measure based on those data. In the most common example,  y is the log of the sample OR for the occurrence of an event.

The theory associated with the conventional random‐effects analysis assumes that the distribution of y_j_ can be adequately approximated by $N(\theta_{j},\sigma_{j}^{2})$. In the resulting normal‐normal model, the marginal distribution of y_j_ is $N(\theta_{j},\sigma_{j}^{2}+\tau^{2})$. The conventional approach then estimates θ by a weighted mean of the y_j_ with inverse‐variance weights. Theory yields the optimal weights, $1/(\sigma_{j}^{2}+\tau^{2})$, but both $\sigma_{j}^{2}$ and τ ^2^ are unknown. Thus, applications use $s_{j}^{2}$ instead of $\sigma_{j}^{2}$ and estimate τ ^2^, producing the weights $w_{j}=1/(s_{j}^{2}+{\hat{\tau }}^{2})$. A key assumption is that one can substitute $s_{j}^{2}$ for $\sigma_{j}^{2}$ without allowing for its variability. Despite its documented shortcomings,(11, 12) this approach has remained an acceptable part of research on random‐effects meta‐analysis, and it serves as the basis for most applications. For the log‐odds‐ratio, for example, the logit transformation yields the logit‐normal‐normal model: if n_jC_ and y_jC_ denote the sample size and number of events in the control group of study  j and n_jT_ and y_jT_ are the corresponding data in the treatment group, the log of the sample OR is 

$$\begin{array}{ccc} y_{j} & =\log(p_{jT}/(1-p_{jT}))-\log(p_{jC}/(1-p_{jC})) & \\ & =\text{logit}(p_{jT})-\text{logit}(p_{jC}), \end{array}$$

where p_jT_ = y_jT_/n_jT_ and p_jC_ = y_jC_/n_jC_, which results in the estimate of the log‐odds‐ratio

$$y_{j}=\log\left(\frac{y_{jT}/(n_{jT}-y_{jT})}{y_{jC}/(n_{jC}-y_{jC})}\right),$$

and the customary estimate of its variance is

$$s_{j}^{2}=\frac{1}{y_{jT}}+\frac{1}{n_{jT}-y_{jT}}+\frac{1}{y_{jC}}+\frac{1}{n_{jC}-y_{jC}}$$

(though in common use, these estimates are biased^13^); if the 2 × 2 table contains one zero cell, 0.5 is usually added to all four cells; if the 2 × 2 table contains two zero cells, study  j is omitted from the analysis. The assumption of a normal distribution for y_j_ in typical finite samples, however, has little empirical support, and correlation between y_j_ and $s_{j}^{2}$ is a potential source of bias.

Similarly, the log of the sample RR,

$$y_{j}=\log(p_{jT})-\log(p_{jC}),$$

results in the estimate

$$y_{j}=\log\left(\frac{y_{jT}/n_{jT}}{y_{jC}/n_{jC}}\right),$$

and the customary estimate of its variance is

$$s_{j}^{2}=\frac{1}{y_{jT}}-\frac{1}{n_{jT}}+\frac{1}{y_{jC}}-\frac{1}{n_{jC}}$$

(as above, these estimates are biased^14^).

## 3 BINOMIAL GLMMs 

For the important class of applications in which the individual outcome is binary, the available data from each study usually include the sample size and number of events in each group. Then, a likelihood‐based analysis can avoid the assumptions and approximations of using a normal distribution for y_j_. For simplicity, we consider only GLMMs based on the summary data available from K 2 × 2 tables (ie, the numbers of events Y_jC_ and Y_jT_ out of n_jC_ and n_jT_ binomial trials, with probability of an event π_jC_ and π_jT_, respectively). In their discussion of these multilevel models, Turner et al^15^ use the term  individual data methods  when the individual subjects' data are binary (and  summary data methods  when the measure of effect is the sample log‐odds‐ratio), but their analyses use the data from 2 × 2 tables.

This section reviews logistic linear mixed models (LMMs), discusses log‐binomial models and their complications, and examines the consequences of using the log‐binomial‐normal model to generate data.

We assume that, given the probabilities π_ji_,

(1) $$\begin{array}{ccc} Y_{ji}|\pi_{ji} & ∼\;\text{Binomial}(n_{ji},\pi_{ji})\;\text{for} & \\ i & =C,T\;\text{and}\;j=1,\text{⋯},K. \end{array}$$

For link function g, the basic GLMM for random‐effects meta‐analysis of treatment versus control is

(2) $$g(\pi_{ji})=\alpha_{j}+(\theta +b_{j})x_{i},$$

where, for study  j, α_j_ is the control group effect; θ is the overall treatment effect; b_j_ is the random treatment effect, representing the departure of Study j's true treatment effect (θ_j_) from θ; and x_i_ is an indicator variable for the treatment group (x_C_ = 0, x_T_ = 1); the b_j_ are independent, and usually b_j_∼N(0,τ ^2^).

The most common link function is the logit transformation g(π)=logit(π)=log(π/(1−π)). The resulting mixed‐effects logistic regression, with log‐odds‐ratio as the effect measure, belongs to the class of GLMMs, discussed in meta‐analysis by Turner et al^15^ and Stijnen et al.^16^ We also consider the log link, which corresponds to the log‐risk‐ratio. Meta‐regression models expand Equation 2 to include study‐level covariates.

For actual analyses and for simulation studies of log‐odds‐ratios, the two‐level logit‐binomial‐normal model is attractive, for several reasons: log‐odds is compatible with binomial likelihoods, the values of θ are not bounded, and it is not necessary to rely on asymptotic normality of the sample log(OR). This model is logistic regression with a random effect. Conveniently, it can be fitted by all modern software for GLMMs. Alternatively, one can use a conditional hypergeometric‐normal model, implemented in SAS NLMIXED^16^ and in R in metafor.^17^

### 3.1 Logistic LMMs 

As background for examining the log link function and the log‐binomial models for the log‐risk‐ratio, we review the more‐familiar logistic LMMs.

#### 3.1.1 Fixed‐effects logistic model

The standard fixed‐effects logistic model does not account for heterogeneity of the ORs between studies. Assuming a binomial distribution in the two arms, the model is (j = 1,…,K)

(3) $$\log\left(\frac{\pi_{ji}}{1-\pi_{ji}}\right)=\alpha_{j}+\theta x_{i},\;$$

where the α_j_ are fixed control group effects (usually regarded as nuisance parameters) and θ is the overall log‐odds‐ratio. The K + 1 parameters of this model can be estimated using maximum likelihood (ML).

#### 3.1.2 Logistic LMMs 

A basic mixed‐effects logistic regression model fits fixed effects for the studies' control groups and accounts for heterogeneity in ORs among studies. Given the binomial distributions in the two arms (1), the model is (j = 1,…,K)

(4) $$\log\left(\frac{\pi_{ji}}{1-\pi_{ji}}\right)=\alpha_{j}+(\theta +b_{j})x_{i},$$

where the α_j_ are fixed control group effects (usually regarded as nuisance parameters), θ is the overall log‐odds‐ratio, b_j_∼N(0,τ ^2^) are random effects, and τ ^2^ is the between‐study variance. The fixed study‐specific intercepts α_j_ have to be estimated, along with θ and τ ^2^. These K + 2 parameters are estimated iteratively, using marginal quasi‐likelihood, penalized quasi‐likelihood, or a first‐ or second‐order Taylor‐expansion approximation. A fixed‐effect meta‐analysis corresponds to τ ^2^ = 0.

As K becomes large, it may be inconvenient, even problematic, to have a separate α_j_ for each study. One can replace those fixed effects with random effects α + u_j_, centered at α:

(5) $$\log\left(\frac{\pi_{ji}}{1-\pi_{ji}}\right)=\alpha +u_{j}+(\theta +b_{j})x_{i}.$$

As before, θ is the overall log‐odds‐ratio, and b_j_∼N(0,τ ^2^). Now u_j_∼N(0,σ ^2^), and u_j_ and b_j_ can be correlated: Cov(u_j_,b_j_) = ρστ. Heterogeneity of log‐odds in the control groups is represented by the variance σ ^2^, and in the treatment groups, by σ ^2^ + 2ρστ + τ ^2^. In contrast, the conventional REM, which works with the sample log‐odds‐ratios, involves only a single between‐study variance, τ ^2^. Turner et al 15 point out that ρ should be estimated. Assuming that ρ = 0 would impose the potentially inappropriate restriction that the variation among trials for control groups (σ ^2^) must be less than or equal to the variation among trials for treatment groups (σ ^2^ + τ ^2^).

Estimation of α, μ, σ ^2^, τ ^2^, and ρ is similar to estimation of the parameters in Model (4) (Turner et al^15^). The related bivariate logistic‐normal model discussed by Stijnen et al^16^ assumes a bivariate normal distribution for log‐odds in the two arms of each study.

### 3.2 Log‐binomial models

This section examines the use of the log link function in binomial GLMs and GLMMs.

#### 3.2.1 Fixed‐effects log‐binomial model

The log‐binomial model is a constrained GLM with the log link function  

(6) $$\begin{array}{ccc} \log(\pi_{jC}) & =\alpha_{j}\leq 0, & \\ \log(\pi_{jT}) & =\alpha_{j}+\theta \leq 0. \end{array}$$

As in the logistic model, the α_j_ are nuisance parameters; here,  θ is the overall log‐risk‐ratio. The linear constraints in (6) guarantee that π_jC_ < 1 and π_jT_ < 1. Likelihood‐based methods must ensure that the estimates satisfy these restrictions. They may cause convergence problems, but neglecting them may lead to wrong estimates. Luo et al^18^ provide a brief review of the existing methods and the requisite R code. They propose an adaptive‐barrier approach to ML estimation that is easily implemented in R, and they compare several methods by simulation. An approach by Donoghoe and Marschner^19^ based on the EM algorithm is implemented in the R package logbin.^20^ Marschner^21^ gives a comprehensive review of contemporary ML and alternative methods, mostly based on unconstrained quasi‐likelihood estimation procedures.

#### 3.2.2 Log‐binomial LMMs 

To the authors' knowledge, no theoretical developments so far have produced log‐binomial mixed models. The main reason, in our opinion, is the restricted parameter space. We now examine this in more detail. In Model (4), mechanically replacing the logit link function by the log link produces the following model for the log‐risk‐ratio:

(7) $$\begin{array}{ccc} \log(\pi_{ji}) & =\alpha_{j}+(\theta +b_{j})x_{i},\;b_{j}∼N(0,\tau^{2}); & \\ j & =1,\text{⋯},K;\;i=C,T. \end{array}$$

Here, the $\alpha_{j}=\log(\pi_{jC})<0$, but the restriction π_jT_ < 1 implies that

(8) $$b_{j}<-\log(\pi_{jC})-\theta ,\;j=1,\text{⋯},K.$$

The probability that b_j_ satisfies this restriction is $\Phi ((-\log(\pi_{jC})-\theta )/\tau )$, where Φ is the cumulative distribution function of the standard normal distribution. Thus, as written, the model in Equation 7 is improper. If π_jC_ and θ are very small, this probability may be almost 1, so that the restriction has little impact; but for moderate π_jC_ and/or larger values of θ, it becomes a serious issue. As an example, for τ ^2^ = 1 and θ = 0, the probability is 0.989 when π_jC_ = 0.1 and 0.886 when π_jC_ = 0.3, decreasing to 0.904 and 0.581 when θ = 1. These probabilities apply to an individual b_j_. For τ ^2^ = 1,θ = 0, and π_jC_ = 0.1, for example, the probability that all K of the b_j_ satisfy the restriction is (0.989)^K^, which equals 0.948 when K = 5, 0.898 when K = 10, and 0.807 when K = 20. To summarize, restriction (8) is not compatible with  model (7), which needs to be replaced by an appropriate model. A simple modification by Warn et al,^22^ in the context of Bayesian modeling of RR and RD, replaces Equation 7 by

(9) $$\begin{array}{ccc} \log(\pi_{ji}) & =\alpha_{j}+\left(\theta +b_{j}^{U}\right)x_{i},\;b_{j}∼N(0,\tau^{2}); & \\ b_{j}^{U} & =\min(-\log(\pi_{jC})-\theta ,b_{j});\;j=1,\text{⋯},K;\\ i & =C,T. \end{array}$$

This introduces a point mass of probability 1 − Φ(β) at $c_{j}^{*}$, equal to or just below $c_{j}=-\log(\pi_{jC})-\theta$, for $\beta =(c_{j}^{*}-\theta )/\tau$ and, equivalently, imputes the values of π_jT_ at or just below 1. Rhodes et al^23^ use this model for Bayesian analysis of inconsistency in the Cochrane database.

An alternative truncates the normal distribution of random effects (b_j_) from the right at $A_{j}=-\log(\pi_{jC})-\theta$. We denote the normal distribution N(μ,σ ^2^) truncated from above at A by TN(μ,σ ^2^,A). Then the model is

(10) $$\begin{array}{cc} \log(\pi_{ji}) & =\alpha_{j}+(\theta +b_{j})x_{i},\;b_{j}∼TN(0,\tau^{2},-\log(\pi_{jC})-\theta );\\ j & =1,\text{⋯},K;\\ i & =C,T. \end{array}$$

Instead of implementing the restriction in Equation 8, an impossible task, both models distort the distribution of θ_j_. Restrictions depending on the values of π_jC_ and θ make both models very artificial.

In both models, the expected value of the log‐risk‐ratio, E(θ_j_), no longer equals θ, the overall log‐risk‐ratio in Equation 7. The expected value of θ_j_ in model (9) is

$$\begin{array}{ccc} E(\theta_{j}) & =\theta -\frac{1}{\sqrt{2\pi \tau^{2}}}\int_{c^{*}}^{\infty }x\exp(-{\left((x-\theta )/\tau \right)}^{2})dx & \\ & \;+c^{*}(1-\Phi (\beta ))<\theta , \end{array}$$

where ϕ is the probability density function of the standard normal distribution and $c^{*}=-\log(\pi_{jC})$. A parallel calculation yields $E(\theta_{j}^{2})$ and hence var(θ_j_).

Next, we determine the corresponding mean and variance of θ_j_ in model (10). For X having a TN(μ,τ ^2^,A) distribution, let β = (A − μ)/τ. Then (see, eg, Barr and Sherrill^24^), 

$$\begin{array}{ccc} E(X) & =\mu -\tau \frac{\phi (\beta )}{\Phi (\beta )}\;\text{and}\;\; & \\ \mathrm{Var}(X)\; & =\tau^{2}\left[1-\beta \frac{\phi (\beta )}{\Phi (\beta )}-{\left(\frac{\phi (\beta )}{\Phi (\beta )}\right)}^{2}\right]. \end{array}$$

In our context, $A=-\log(\pi_{Cj})$, μ = θ, and $\beta =(-\log(\pi_{Cj})-\theta )/\tau$. Therefore, the mean of θ_j_ is less than θ, and it decreases with increasing π_jC_. The variance of θ_j_ is noticeably smaller than τ ^2^, decreasing as π_jC_ increases. This model is also clearly not satisfactory.

Importantly, in both models, the expected values of the log‐risk‐ratios θ_j_ depend on the individual values of π_jC_, making the meta‐analysis of the θ_j_ rather pointless.

In Section 3.3, we consider in more detail what happens when model  (7) is used and the restrictions are neglected. As we shall see, this mistake results in considerable biases. Overall, we find the log‐binomial LMMs with fixed α_j_ not suitable for modeling the RR.

The analog of model  (5), with random effects for the control groups, is

(11) $$\begin{array}{ccc} \log(\pi_{ji}) & =\alpha +u_{j}+(\theta +b_{j})x_{ji},\;b_{j}∼N(0,\tau^{2}),\; & \\ & u_{j}∼N(0,\sigma^{2}),\;\text{and}\;Cov(u_{j},b_{j})=\rho \sigma \tau . \end{array}$$

This model involves even more restrictions:

(12) $$\begin{array}{ccc} & u_{j}<-\log(\pi_{jC})-\alpha ,\;b_{j}+u_{j}<-\log(\pi_{jC})-\alpha -\theta , & \\ & j=1,\text{⋯},K, \end{array}$$

so it also is not suitable.

To summarize, we do not think that a GLMM with the log link is a feasible option for modeling relative risk.

### 3.3 Generating data from the log‐binomial LMM

In this section, we discuss the consequences of using the log‐binomial‐normal mixed model, Equation 7, to generate data, and we use a small simulation study for illustration.

#### 3.3.1 Practicalities

In practice, the studies in a meta‐analysis come from a systematic review, bringing with them the underlying pairs of event probabilities, (*π*_*jC*_,*π*_*jT*_). For REMs, it is convenient to regard the (*π*_*jC*_,*π*_*jT*_), and hence the $\left(\log\pi_{jC},\log\pi_{jT}\right)$, as a sample from some bivariate distribution. We can also approach the joint distribution of (*π*_*jC*_,*π*_*jT*_) via the marginal distribution of *π*_*jC*_ and the conditional distribution of *π*_*jT*_ given *π*_*jC*_ and a value of *θ*. Thus, to obtain data from the log‐binomial LMM for meta‐analysis of log‐risk‐ratio, we can choose values of *π*_*jC*_, generate study effects *θ*_*j*_ from *N*(*θ*,*τ* ^2^), calculate the $\pi_{jT}=\pi_{jC}\exp(\theta_{j})$, generate observations *Y*_*jC*_ from the Binomial(*n*_*jC*_,*π*_*jC*_)  distributions, and generate observations *Y*_*jT*_ from the Binomial(*n*_*jT*_,*π*_*jT*_) distributions (this approach parallels a common method of generating data for meta‐analyses of ORs). 

However, this process may produce values of *π*_*jT*_ > 1. As a remedy, one has two practical options: either impute values of *π*_*jT*_ at or slightly below 1 or reject values of *θ*_*j*_ that are too large and generate replacement values of *θ*_*j*_. The first option is equivalent to using model (9), and the second option (rejection sampling) is equivalent to truncating the normal distribution of random effects (*b*_*j*_) as in model (10). Both options introduce bias; that is, *E*(*θ*_*j*_) no longer equals *θ*, the overall log‐risk‐ratio in Equation 7. The first option appears to be more popular in meta‐analysis. IntHout et al^25^ use it in their simulations. The second option seems uncommon, but many authors who use simulation in meta‐analysis do not report details of implementation. Some authors use truncate  but create a point mass (eg, Panityakul et al^26^). Pedroza and Truong^27^ use truncation in simulating risk difference in multicenter trials. Both options aim to approximate the actual situation, in which *π*_*jC*_ < 1 and *π*_*jT*_ < 1. The basic difficulty lies in using a normal distribution for the random effects. A different approach is required to obtain unbiased inference, or the bias needs to be estimated and eliminated.

#### 3.3.2 Simulation study

To evaluate the size of these biases in conventional random‐effects meta‐analysis (ie, the log‐normal‐normal model, Section 2), we conducted a small simulation study of the two options for generating data from a log‐binomial LMM. We used equal sample sizes *n*_*jC*_ = *n*_*jT*_ = *n*/2 and the same value of *π*_*C*_ for all studies. We set *K* = 5,10,20; *n* = 100,200,500; *θ* = −1.5,−1,−0.5,0,0.5,1,1.5; *π*_*jC*_ = 0.1 or 0.3; and *τ* ^2^ = 0.1 and 1. If a  *study*  had *Y*_*T*_ + *Y*_*C*_ = 0 or *n*, we followed customary practice by discarding it and reducing *K* accordingly. We estimated *τ* ^2^ by three methods: DerSimonian‐Laird,^28^ Mandel‐Paule,^29^ and restricted maximum likelihood (REML). From 1000 replications, we studied estimation of *τ* ^2^ and *θ* and the coverage of confidence intervals for *θ* based on the normal approximation.

**Figure 1:**
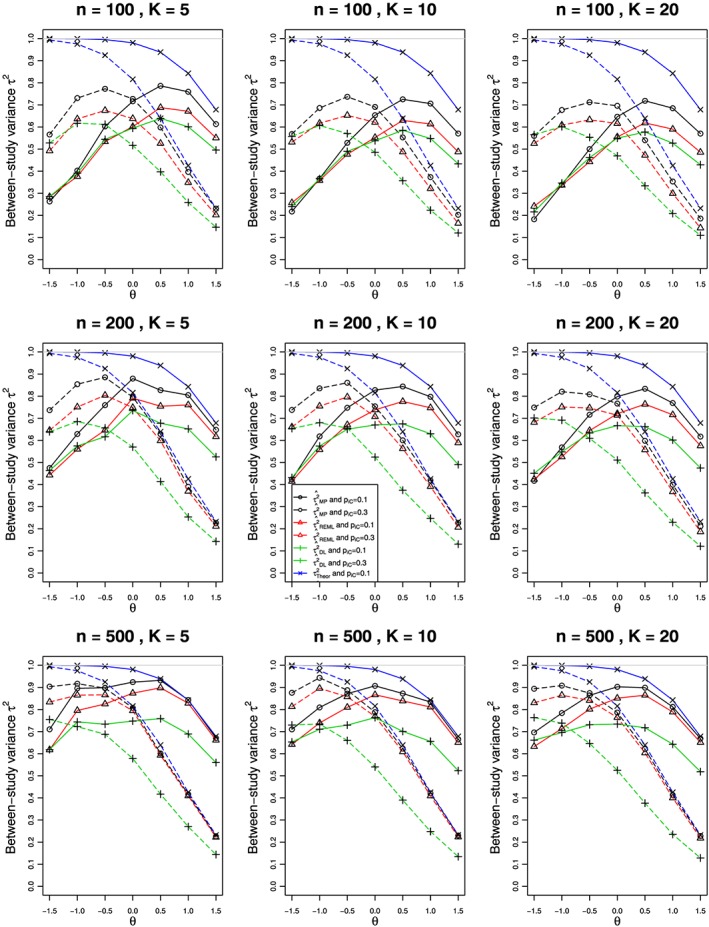
Relation of estimates of the between‐studies variance (τ ^2^) to the overall log‐risk‐ratio (θ) in K studies, each of total sample size n, when data come from the binomial‐normal model with point mass for τ ^2^ = 1 and π_jC_ = 0.1 (solid lines) and 0.3 (dashed). The Mandel‐Paule (circle), REML (triangle), and DerSimonian‐Laird (plus) estimators are compared with the true variance (cross). Light gray line at 1 [Colour figure can be viewed at wileyonlinelibrary.com]

**Figure 2:**
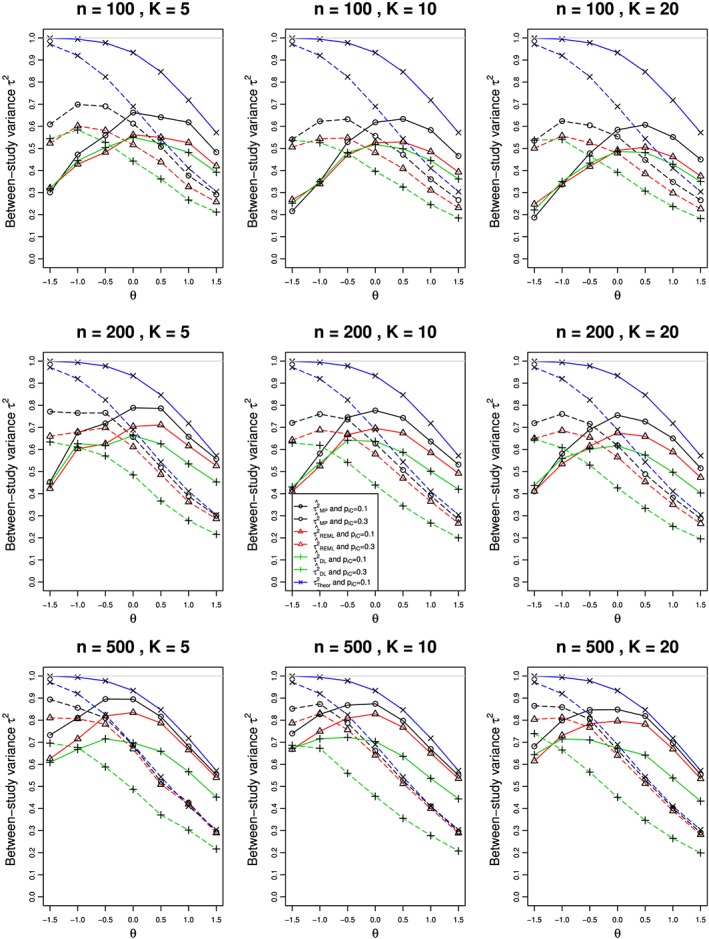
Relation of estimates of the between‐studies variance τ ^2^ to the overall log‐risk‐ratio (θ) in K studies, each of total sample size n, when data come from the binomial‐normal model with truncation for τ ^2^ = 1 and π_jC_ = 0.1 (solid lines) and 0.3 (dashed). The Mandel‐Paule (circle), REML (triangle), and DerSimonian‐Laird (plus) estimation methods are compared with the true variance (cross). Light gray line at 1 [Colour figure can be viewed at wileyonlinelibrary.com]

Figures 1 and 2 show the results for estimation of *τ* ^2^ (ie, var(*θ*_*j*_)) when *τ* ^2^ = 1. Similar results for *τ* ^2^ = 0.1 appear in Figures S1 and S2 (Appendix B.1). The substantial biases (usually negative) in estimation of *τ* ^2^ for both options have two sources. For the larger values of *θ*, they arise from the restriction *π*_*jT*_ < 1, which has greater impact for *π*_*jC*_ = 0.3 than for *π*_*jC*_ = 0.1. As the traces for the theoretical value of *τ* ^2^ show, this source of bias plays a steadily decreasing role as *θ* decreases from 1.5 to −1.5. For the smaller (ie, more negative) values of *θ*, the source of the bias is progressively small values of *π*_*jT*_  as *θ* becomes more negative. For example, when *π*_*jC*_ = 0.1, and *θ* = −0.5, the median value of *π*_*jT*_ is 0.0607, for *θ* = −1, the median value is 0.0368, and for *θ* = −1.5, it is 0.0223. For sparse data, the distribution of log(risk) and hence log(RR) is not well approximated by a normal distribution. It is well known that the standard REM does not perform well in these circumstances.^30^

In relation to *θ*, the point‐mass option (Figure 1) has similar patterns of bias in ${\hat{\tau }}^{2}$ for the three methods as *K* increases and as *n* increases. The MP method consistently has the smallest bias, followed by REML and then DL. As *K* increases, the patterns for each *n* change little. As *n* increases, the traces for each *K* move closer to 1, and the trace for REML moves closer to that for MP. In contrast, the traces for DL generally move farther away from the other estimators.

For the truncation option, the plots of bias in ${\hat{\tau }}^{2}$ versus *θ* (Figure 2) are qualitatively similar to those for the point‐mass option (Figure 1), with several main differences. For *π*_*jC*_ = 0.1 and each combination of *n* and *K*, the biases are larger than those in Figure 1, especially for *θ* ≥ 0. For *π*_*jC*_ = 0.3 and *θ* > 0, the slopes are not as steep, and the biases at *θ* = 1.5 are not as large, as in Figure 1.

**Figure 3:**
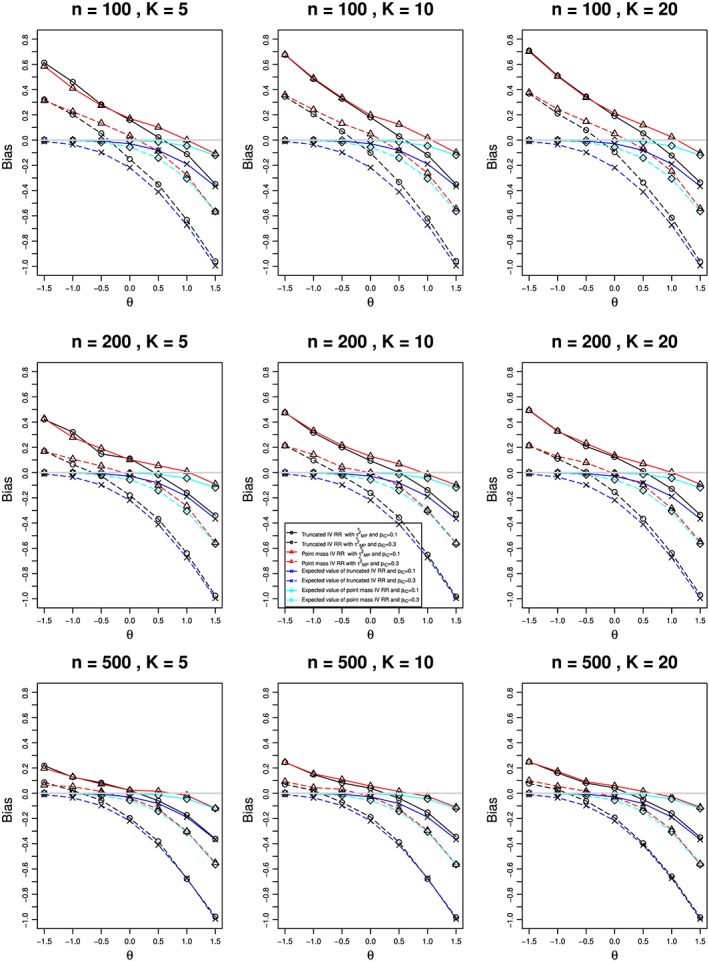
Relation (to the overall log‐risk‐ratio, θ) of bias in the conventional method of estimating the log‐relative‐risk, θ, in the binomial‐normal model from K studies, each of total sample size n, with truncation (circle) or point‐mass (triangle) option, when τ ^2^ (true value, τ ^2^ = 1) is estimated by the Mandel‐Paule method, compared with true bias from truncation (cross) and point mass (diamond). π_jC_ = 0.1 (solid lines) and 0.3 (dashed). Light gray line at 0 [Colour figure can be viewed at wileyonlinelibrary.com]

Biases in estimating *θ* are almost the same for the three methods of estimating *τ* ^2^. Therefore, Figure 3 shows the results for the Mandel‐Paule method and, for comparison, the theoretical expectations. For both options and both values of *π*_*jC*_, the bias in $\hat{\theta }$ is strongly related to *θ*. When *π*_*jC*_ = 0.1, the two options produce the same bias for *θ* ≤ 0: positive at *θ* = 0 and roughly linear in *θ*, with negative slope, for *θ* < 0 (we expect the restriction *π*_*jT*_ < 1 to have little impact). For *θ* > 0, the traces for the two options diverge; the point‐mass option has bias of relatively small magnitude, and the truncation option has increasingly negative bias as *θ* increases. When *π*_*jC*_ = 0.3, both traces show substantial curvature. For *θ*≤ − 0.5, truncation often produces smaller (and positive) bias than the point‐mass option, but for *θ* ≥ 0, its bias is negative and considerably larger in magnitude. These patterns change little with *K* and only slightly with *n*.

In summary, neither the point‐mass option nor the truncation option responds satisfactorily to *b*_*j*_ sampled from a random‐effects distribution that produces *π*_*jT*_ > 1. The resulting biases in estimating *θ* and *τ* ^2^ are often unacceptably large. Our choice of *τ* ^2^ = 1 as the true value may have magnified the biases, but it serves to illustrate the difficulties, and the same general patterns in estimating *τ* ^2^ are present when *τ* ^2^ = 0.1. The biases seen in this small simulation raise questions about the results of numerous meta‐analyses that have employed the REM for RRs. We explore this further in Section 6.

## 4 BB MODEL

In this section, we explore the main alternative to the binomial GLMMs, BB regression, and its application to meta‐analysis of RRs.

### 4.1 The BB distribution

The BB distribution arises as a mixture of binomial distributions, Binom(*n*,*p*), according to a beta distribution for *p*. If *Y*∼Binom(*n*,*p*) and *p*∼Beta(*α*,*β*), then, unconditionally, *Y* follows a BB distribution with parameters *n*, *α*, and *β* (Johnson et al,^31^, p.270). It is convenient to parametrize this distribution as BetaBinom(*n*,*π*,*ρ*), where *π* = *α*/(*α* + *β*), *ρ* = 1/(*α* + *β* + 1), *α* > 0, and *β* > 0. Then, the beta distribution has mean *π* and variance *π*(1 − *π*)*ρ*, and

(13) E(Y)=nπ,Var(Y)=nπ(1−π)(1+(n−1)ρ),

which shows overdispersion relative to Binom(*n*,*π*). The distribution of the sum of *n* Bernoulli(*π*) random variables with intra‐cluster correlation (ICC) *ρ* has the same mean and variance, but its actual shape may be very different.^32^

### 4.2 BB regression

Like the conventional REM and the binomial GLMMs, BB regression is a two‐stage REM. Assume that, as in a randomized controlled trial, the treatment and control groups of each study are independent, and that within the two groups, conditional on the probabilities, the numbers of events follow binomial distributions. Allowing beta‐distributed variation of the probabilities among the studies, the resulting marginal distributions are BB distributions. If (for simplicity) they have the same *ρ* for both groups and all studies, then

(14) $$\begin{array}{ccc} Y_{jC} & ∼\text{BetaBinom}(n_{jC},\pi_{C},\rho )\;\text{and} & \\ Y_{jT} & ∼\text{BetaBinom}(n_{jT},\pi_{T},\rho ). \end{array}$$

That is, as in the binomial model, the two groups differ only on *π* (and *n*). The corresponding beta distributions are

(15) $$p_{ji}∼\text{Beta}(\pi_{i}(\rho^{-1}-1),(1-\pi_{i})(\rho^{-1}-1)).$$

Importantly, because the GLMMs and BB regression make very different distributional assumptions about the random effects, their results may differ. Bakbergenuly and Kulinskaya^33^ considered meta‐analysis of ORs under a BB model. A more general model uses a bivariate beta distribution, and therefore may have different values of *ρ* in the two groups, and also correlation between the groups.^34^

Like GLMMs, the BB model can incorporate a matrix of covariates **X** by using an appropriate link function *g* for the effect measure of interest, so that *g*(*π*) = **X** *θ* for a vector of unknown parameters *θ*.

For BB meta‐analysis,

$$g(\pi_{i})=\alpha +x_{i}\theta ;$$

as before, *x*_*i*_ is a treatment indicator, and *θ* is a treatment effect. Thus, BB regression yields a REM in which *θ* determines the association between *p*_*jT*_ and *p*_*jC*_ (through the link function).

Most, if not all, BB regression programs use the logit link,(35, 36) so the probabilities *π* = expit(**X** *θ*). The logit link function guarantees that the probabilities *π* lie in the interval (0,1). The log link encounters the same complications as in the log‐binomial model, because the estimation process needs to incorporate the constraint **X** *θ*<0 . We are not aware of any theoretical work for this log‐beta‐binomial model.

### 4.3 Using standard BB regression for RR 

In a single study, the RR can be estimated as the ratio ${\hat{\pi }}_{T}/{\hat{\pi }}_{C}$ of the ML estimators of *π*_*T*_ and *π*_*C*_ or from the estimated logits obtained by using a BB regression with the logit link.

The likelihood for the BB model (14) is

(16) ∏j=1K∏i=12njiYjiBeta(πi(ρ−1−1)+Yji,(1−πi)(ρ−1−1)+nji−Yji)Beta(πi(ρ−1−1),(1−πi)(ρ−1−1)),

where Beta(*u*,*v*)  is the beta function. The parameters *π*_*T*_, *π*_*C*_, and *ρ* can be estimated by maximizing the log‐likelihood. This process may encounter computational problems because the BB distribution does not belong to an exponential family.^37^ML estimation requires numerical methods such as the Newton‐Raphson method. The approximate covariance matrix of the parameter estimates is obtained by evaluating the inverse of the Hessian matrix at those estimates.

In R, the package *bbmle* provides a program for maximizing the BB likelihood.^38^ General BB regression with the logit link function is implemented in a number of R packages, including *gamlss* 39 and *hglm*.^36^ The use of the SAS procedure NLMIXED is explained in Martinez et al.^37^

From the MLEs of *π*_*T*_ and *π*_*C*_ and estimates of their variances $Var({\hat{\pi }}_{T})$ and $Var({\hat{\pi }}_{C})$ (say, from *bbmle*), the delta method yields an approximation for the variance of log(RR):

(17) $$\;\mathrm{Var}(\log(RR))\approx {\left[\frac{1}{{\hat{\pi }}_{T}}\right]}^{2}\mathrm{Var}({\hat{\pi }}_{T})+{\left[\frac{1}{{\hat{\pi }}_{C}}\right]}^{2}\mathrm{Var}({\hat{\pi }}_{C}).$$

Similarly, when using the logit link, output from BB regression (say, *gamlss*) provides estimates of the log‐odds ${\hat{\eta }}_{C}=\hat{\alpha }$ and ${\hat{\eta }}_{T}=\hat{\alpha }+\hat{\theta }$ and their standard errors. To obtain the estimate of the RR, the expit transformation yields the estimated probabilities

$${\hat{\pi }}_{C}=\frac{\exp(\hat{\alpha })}{1+\exp(\hat{\alpha })}\;\text{and}\;{\hat{\pi }}_{T}=\frac{\exp(\hat{\alpha }+\hat{\theta })}{1+\exp(\hat{\alpha }+\hat{\theta })}.$$

Then, log(RR) is given by $\log({\hat{\pi }}_{T}/{\hat{\pi }}_{C})$ with variance approximated by the delta method:

(18) $$\begin{array}{ccc} \;\mathrm{Var}(\log(RR)) & \approx \mathrm{Var}\left(\log\left(\frac{\exp({\hat{\eta }}_{T})}{1+\exp({\hat{\eta }}_{T})}\right)\right. & \\ & \;\left.-\log\left(\frac{\exp({\hat{\eta }}_{C})}{1+\exp({\hat{\eta }}_{C})}\right)\right)\\ & ={\left[\frac{1}{1+\exp({\hat{\eta }}_{T})}\right]}^{2}\;\mathrm{Var}({\hat{\eta }}_{T})\\ & \;+{\left[\frac{1}{1+\exp({\hat{\eta }}_{C})}\right]}^{2}\mathrm{Var}({\hat{\eta }}_{C}). \end{array}$$

The overdispersion in the BB model may be parametrized in various ways: *bbmle* estimates *γ* = (1 − *ρ*)/*ρ*, and *gamlss* estimates γ=log(ρ). In Supporting Information (Appendix A), we provide R functions for using *bbmle* and *gamlss* for meta‐analysis of RR.

### 4.4 Conventional meta‐analysis of RRs under the BB model

Conventional meta‐analysis calculates the sampleRRs, ${\hat{\psi }}_{j}={\hat{\pi }}_{jT}/{\hat{\pi }}_{jC}$, and their logarithms, ${\hat{\theta }}_{j}$, and uses inverse‐variance weights based on estimated variances of the ${\hat{\theta }}_{j}$. To obtain an unbiased (to *O*(*n* ^−2^)) estimate of *θ*_*j*_ and an unbiased (to *O*(*n* ^−3^)) estimate of $Var({\hat{\theta }}_{j})$ under a binomial distribution, Pettigrew et al^14^ add 1/2 to the number of events and the total in each group:

(19) $${\hat{\theta }}_{j}=\log\left(\frac{Y_{jT}+1/2}{n_{jT}+1/2}\right)-\log\left(\frac{Y_{jC}+1/2}{n_{jC}+1/2}\right);$$

we retain this estimate. When *Y*_*jC*_ and *Y*_*jT*_ have BB distributions, Equation 14, the approximate variance of ${\hat{\theta }}_{j}$, obtained via the delta method, is

(20) $$\begin{array}{ccc} \;\mathrm{Var}\;({\hat{\theta }}_{j}) & \approx \frac{1-\pi_{T}}{n_{jT}\pi_{T}}(1+(n_{jT}-1)\rho ) & \\ & \;+\frac{1-\pi_{C}}{n_{jC}\pi_{C}}(1+(n_{jC}-1)\rho ). \end{array}$$

Substituting the variances of the ${\hat{\pi }}_{i}$ from the line above (13) yields the same variance as in Equation 17. Setting *ρ* = 0 in (20) yields the within‐study variance of ${\hat{\theta }}_{j}$ for binomially distributed data, so under the BB model (*ρ* > 0), this variance is inflated. In a direct parallel, in the conventional REM, compared with the fixed‐effect model, the variances of the ${\hat{\theta }}_{j}$ are inflated by an additive variance component, *τ* ^2^. Thus, the BB model is similar to the conventional REM, but the variance inflation is multiplicative instead of additive; it is of order *O*(1) and increases with *ρ*; the variance also may be large when *π*_*C*_ or *π*_*T*_ is close to 0.

The conventional REM uses inverse‐variance weights to obtain an estimate of the overall effect. Estimating *π*_*jT*_ and *π*_*jC*_ as in (19) yields the estimated variance

(21) $$\begin{array}{ccc} \;\hat{\mathrm{Var}}({\hat{\theta }}_{j}) & =\left(\frac{1}{y_{jT}+1/2}-\frac{1}{n_{jT}+1/2}\right)(1+(n_{jT}-1)\hat{\rho }) & \\ & \;+\left(\frac{1}{y_{jC}+1/2}-\frac{1}{n_{jC}+1/2}\right)(1+(n_{jC}-1)\hat{\rho }). \end{array}$$

To use these estimated variances, however, we must estimate *ρ*.

### 4.5 Estimation of ρ

To estimate *ρ*, we modify two established methods: a method‐of‐moments estimator based on Cochran's *Q* statistic, similar to the DerSimonian‐Laird^28^ estimator of *τ* ^2^, and an REML estimator. According to Viechtbauer,^40^ these two approaches perform best for estimation of the between‐studies variance *τ* ^2^ in the additive REM. We also use a version of the method of Mandel and Paule^29^ to estimate *ρ* from the large‐sample approximation of *Q* by a chi‐squared distribution. All three methods were proposed by Kulinskaya and Olkin,^41^ but they have not previously been explored by simulation. We refer to these estimators as ${\hat{\rho }}_{\text{MoM}}$, ${\hat{\rho }}_{\text{REML}}$, and ${\hat{\rho }}_{\text{MP}}$, respectively. However, in finite samples the chi‐squared distribution is a poor approximation to the distribution of the *Q* statistic,^42^ and we propose a new method for point and interval estimation of *ρ* based on inverting the modified Breslow‐Day (BD) test,^43^ ${\hat{\rho }}_{\text{BD}}$. Bakbergenuly and Kulinskaya^33^ proposed a similar method in meta‐analysis of ORs. The detailed derivations for these four estimators of *ρ* are given in Supporting Information (Appendix A1).

### 4.6 Simulation study

To explore the performance of BB methods for meta‐analysis of RRs, we conducted a small simulation study. We used equal sample sizes *n*_*jC*_ = *n*_*jT*_ = *n*/2, the same value of *θ* for all studies, and $\pi_{T}=\pi_{C}\exp(\theta )$ for $\theta <-\log(\pi_{C})$. The data in the treatment and control groups were generated from independent BB distributions. Parallel to Section 3.3.2, we set *K* = 5,10,20; *n* = 100,200,500; *θ* = −1.5,−1.0,−0.5,0,0.5,1,1.5; *π*_*C*_ = 0.1 or 0.3; and *ρ* = 0.1. As in our simulation study for the log‐binomial LMM (Section 3.3.2), when a  *study*  had *Y*_*T*_ + *Y*_*C*_ = 0 or *n*, we discarded it and reduced *K* accordingly.

**Figure 4:**
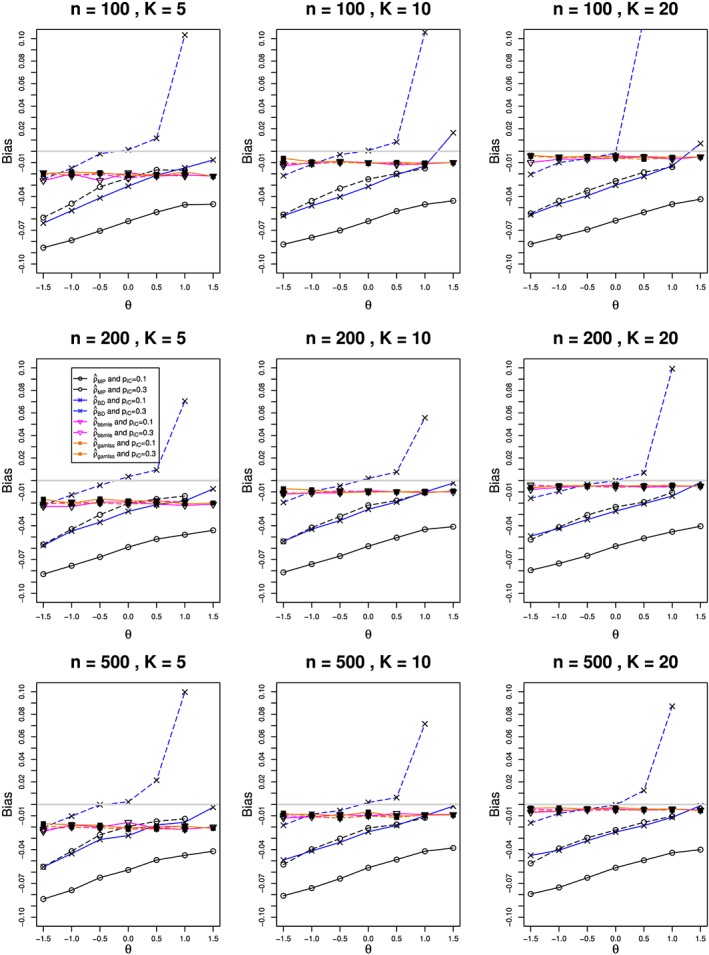
Relation (to the overall log‐risk‐ratio, θ) of bias in estimating ρ from K studies, each of total size n, in the beta‐binomial model for ρ = 0.1 and π_C_ = 0.1 (solid lines) and 0.3 (dashed). The methods are Mandel‐Paule (circle), Breslow‐Day (cross), bbmle (reverse triangle), and gamlss (filled square). Light gray line at 0 [Colour figure can be viewed at wileyonlinelibrary.com]

**Figure 5:**
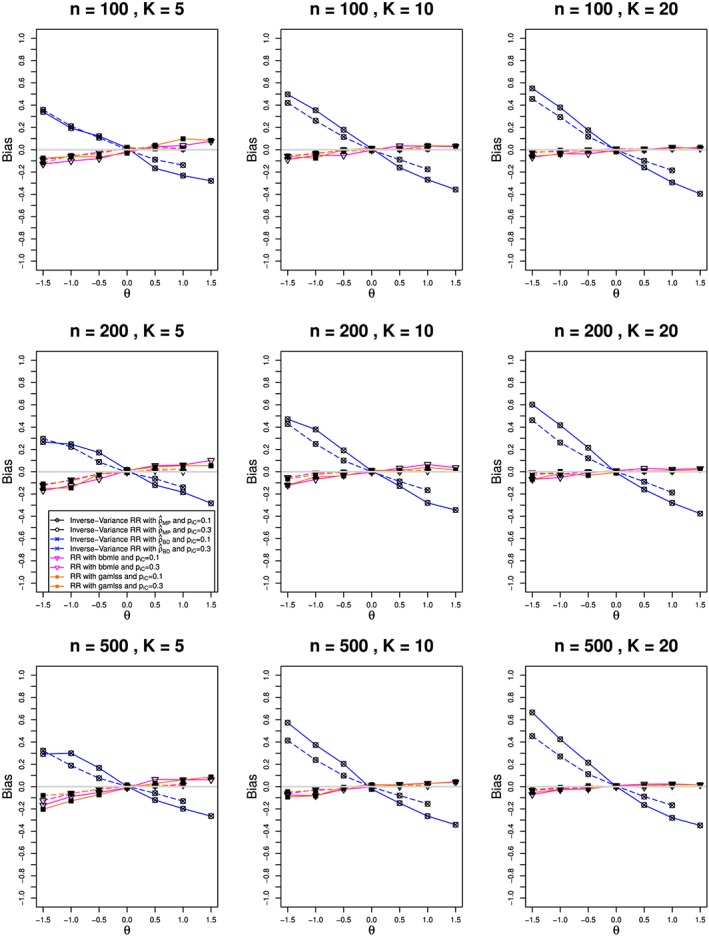
Bias in estimating the overall log‐risk‐ratio, θ, from K studies, each of total size n, in the beta‐binomial model for ρ = 0.1 and π_C_ = 0.1 (solid lines) and 0.3 (dashed). The log‐relative‐risk is estimated by using inverse‐variance weights. The methods for estimation of ρ are Mandel‐Paule (circle), Breslow‐Day (cross), bbmle (reverse triangle), and gamlss (filled square). Light gray line at 0 [Colour figure can be viewed at wileyonlinelibrary.com]

**Figure 6:**
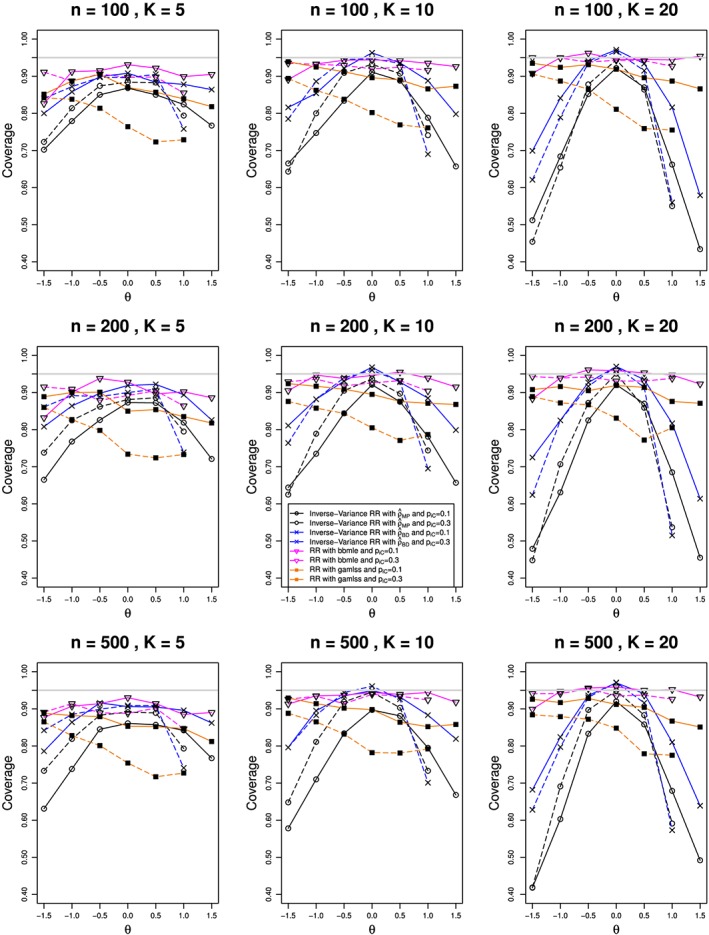
Coverage of the overall log‐risk‐ratio, θ, from K studies, each of total size n, in the beta‐binomial model for ρ = 0.1 and π_C_ = 0.1 (solid lines) and 0.3 (dashed). The log‐relative‐risk is estimated by using inverse‐variance weights. The methods for estimation of ρ are Mandel‐Paule (circle), Breslow‐Day (cross), bbmle (reverse triangle), and gamlss (filled square). Light gray line at 0.95 [Colour figure can be viewed at wileyonlinelibrary.com]

We estimated *ρ* by the modified MoM and MP methods, REML, and the BD‐based method. The estimates of *θ* used inverse‐variance weights, and their CIs used the normal approximation. We also included *bbmle* and *gamlss*, the latter with logit link. For all methods, we estimated bias in estimation of *θ* and *ρ*  and coverage of confidence intervals for *θ*. For the nine combinations of *n* and *K*, Figures 4, 5, and 6 plot (versus *θ*) the estimated bias for $\hat{\rho }$ and $\hat{\theta }$  and the coverage of *θ*, respectively. Because the results are almost the same for the MP, MoM, and REML methods, the figures show only the MP results.

For *bbmle* and *gamlss*, the bias in $\hat{\rho }$ does not vary appreciably with *θ*, *π*_*C*_, or *n*. It is roughly −0.02 for *K* = 5, and it approaches 0 as *K* increases. The bias of the other methods is generally negative, unrelated to *n*, and only weakly related to *K*. The relation of those biases to *θ* is roughly linear, with similar positive slopes (except for high positive values with BD when *π*_*C*_ = 0.3). When *π*_*C*_ = 0.1, the bias of MP rises from −0.0855 at *θ* = −1.5 to −0.0386 at *θ* = +1.5; it is closer to 0 by roughly 0.05 when *π*_*C*_ = 0.3. The trace for BD when *π*_*C*_ = 0.1 closely resembles that for MP when *π*_*C*_ = 0.3, and it shifts upward by roughly 0.035 when *π*_*C*_ = 0.3. Since the true value of *ρ* is 0.1, these biases are substantial.

The bias in $\hat{\theta }$ follows two patterns, both of which change little with *N* or *π*_*C*_. One pattern (RR estimated with *bbmle* or *gamlss*) goes linearly from small negative bias at *θ* = −1.5 to small positive bias at *θ* = 1.5, and its magnitude decreases as *K* increases. The other pattern (MP, BD, and other inverse‐variance methods) goes, roughly linearly, from around 0.3 at *θ* = −1.5 to around −0.3 at *θ* = 1.5, when *K* = 5; and the traces become steeper as *K* increases. All of the methods have essentially no bias at *θ* = 0, but otherwise MP and BD underestimate the magnitude of *θ* by about 20*%*. This is due to the effect of transformation bias, which is almost linear in *ρ*. Section 6.2.3 in Bakbergenuly and Kulinskaya^33^ gives a detailed explanation of the similar bias in BB meta‐analysis of log‐odds‐ratio.

The confidence intervals for *bbmle* have the best coverage of *θ*, slightly below the nominal 95*%* when *K* = 5 (particularly when *θ* = −1.5) but differing little from 95*%* when *K* = 10 and *K* = 20. Coverage of the *gamlss* intervals is lower, especially when *π*_*C*_ = 0.3, and it declines steadily as *θ* increases from −1.5 to +1.5; the pattern changes little with *n* or *K*. The inverse‐variance–based methods give close to nominal coverage when *θ* = 0, but they deteriorate rapidly as *θ* departs from 0 in either direction, and that pattern becomes worse as *K* increases (for example, when *K* = 20, coverage of the MP interval is near or below 50*%* at *θ* = −1.5 and *θ* = 1.5, for all three values of *n*). Patterns of bias such as those in Figure 5 would lead us to expect the patterns of coverage in Figure 6.

Overall, the inverse‐variance methods do not help with estimation of RR in the BB model, as it requires the same constraints as the log‐binomial model. BB regression performs much better in estimation of *ρ* and RR, especially for *K* ≥ 10. However, the use of the logit link, as in *gamlss*, does not provide sufficient coverage of RR, and only *bbmle* provides a viable option when the data follow a BB model. We discuss model misspecification issues in Sections 6 and 7.

## 5 EXAMPLES

In this section, we re‐analyze the data from two medical meta‐analyses, comparing two conventional REMs (DL and REML) with the six methods developed for the BB model that we discussed in Section 4. The first meta‐analysis  on the effect of diuretics on pre‐eclampsia^44^ considered the beneficial effects of treatment (ie, the RR of benefit), whereas the second meta‐analysis focused on side‐effects of low‐dosage tricyclic antidepressants in acute depression^45^ (ie, the RR of harm).

### 5.1 Example 1: Effect of diuretics on pre‐eclampsia

A meta‐analysis of nine trials, with a total of 6942 patients, evaluated the effect of diuretics on pre‐eclampsia, a serious complication in pregnancy.^44^ These data (Table 1) have also been analyzed (as ORs) by Thompson and Pocock,^46^ Hardy and Thompson,^47^ Biggerstaff and Tweedie,^48^ Whitehead,^49^ Viechtbauer,^50^ Makambi and Lu,^51^ and Kulinskaya and Olkin.^41^ The incidence of pre‐eclampsia in the control group shows considerable heterogeneity: from 1.9*%* in study 8 to 50.0*%* in study 3. The incidence in over half of the studies is large enough that the OR does not provide a satisfactory approximation for the RR. The study‐level estimates of log(RR) ( ${\hat{\theta }}_{j}$ from (19)) range from −1.33 to +0.91.

**Table 1:** Data from nine trials of diuretics for treatment of pre‐eclampsia in pregnancy. The study‐level estimate of log(RR), ${\hat{\theta }}_{j}$, comes from Equation 19

Study	*Y*_*jC*_	*n*_*jC*_	*Y*_*jC*_/*n*_*jC*_	*Y*_*jT*_	*n*_*jT*_	${\hat{\theta }}_{j}$
1	14	136	0.103	14	131	0.0373
2	17	134	0.127	21	385	−0.8471
3	24	48	0.500	14	57	−0.6947
4	18	40	0.450	6	38	−0.9953
5	35	760	0.046	12	1011	−1.3290
6	175	1336	0.131	138	1370	−0.2619
7	20	524	0.038	15	506	−0.2447
8	2	103	0.019	6	108	0.9083
9	40	102	0.392	65	153	0.0969

For various methods, Table 2 shows the estimated values of *τ* ^2^ for the conventional REM  and of *ρ* in the BB model. In the conventional REM, the DerSimonian‐Laird estimate of *τ* ^2^ is ${\hat{\tau }}_{\text{DL}}^{2}=0.156$, and ${\hat{\tau }}_{\text{REML}}^{2}=0.199$. Viechtbauer^50^ gives Q‐profile confidence intervals for DL, and Hardy and Thompson^47^ give profile‐likelihood confidence intervals for the REML method.

**Table 2:** Point estimates and confidence intervals for τ ^2^, ρ, log‐risk‐ratio (LRR), and risk ratio (RR) in the example of diuretics in pre‐eclampsia^a^

Model	Method	Overdispersion	*L*	*U*	LRR	*L*	*U*	Length	RR	*L*	*U*
		Parameter						of CI			
		*τ* ^2^									
FEM					−0.305	−0.449	−0.161	0.288	0.737	0.638	0.851
REM	DL&IV	0.156	0.049	1.582	−0.437	−0.768	−0.107	0.661	0.646	0.464	0.899
REM	REML&IV	0.199	0.032	0.989	−0.439	−0.799	−0.079	0.720	0.645	0.450	0.924
		*ρ*									
BB	MoM&IV	0.008	0.002	0.093	−0.297	−0.563	−0.032	0.530	0.743	0.570	0.969
BB	REML&IV	0.010	0.001	0.061	−0.305	−0.595	−0.014	0.581	0.737	0.551	0.986
BB	MP&IV	0.016	0.002	0.093	−0.316	−0.644	0.012	0.632	0.729	0.525	1.011
BB	BD&IV	0.019	0.003	0.106	−0.321	−0.668	0.025	0.693	0.725	0.513	1.026
BB	bbmle	0.138	0.077	0.258	−0.257	−1.008	0.495	1.504	0.774	0.365	1.640
BB	gamlss	0.139	0.057	0.300	−0.257	−0.948	0.433	1.381	0.773	0.388	1.542

*Note*. FEM is the fixed‐effect model, REM is the random‐effects model, and BB is the beta‐binomial model. *bbmle* and *gamlss* are beta‐binomial maximum‐likelihood‐based and generalized‐additive‐regression models. The heterogeneity parameter is *τ* ^2^ in REM and *ρ* in the BB model. *L* and *U* denote the lower and upper limits of the 95% confidence intervals (CIs).

For the BB model, six methods provide estimates of *ρ*: 0.138 for *bbmle* and 0.139 for *gamlss*, and 0.008 to 0.019 for the method‐of‐moments, REML, MP, and BD estimators. The separation between results from BB regression methods (bbmle/gamlss) and the inverse‐variance BB methods is in the direction that we would expect from the simulation results on bias in Figure 4, but the magnitude of $\hat{\rho }$ from BB regression methods is greater (perhaps because of the particular mixture of values of the incidence of pre‐eclampsia in the control group).

In *bbmle*, the ML‐based estimates of the means of the two BB distributions are ${\hat{\pi }}_{T}=0.143$ and ${\hat{\pi }}_{C}=0.185$, which result in *RR* = 0.774. The confidence intervals for estimates of probabilities and the overdispersion parameter *γ*( = (1 − *ρ*)/*ρ* = *α* + *β*) are based on the standard errors obtained from the inverse of the observed information matrix. The standard error for the log‐risk‐ratio is obtained by the delta method as a function of *π*_*T*_ and *π*_*C*_, Equation 17.

In *gamlss*, the estimates for probabilities are obtained from the BB regression model with logit link function. The estimates for probabilities obtained by inverting the logit link function, ${\hat{\pi }}_{T}=0.143$ and ${\hat{\pi }}_{C}=0.185$, yield *RR* = 0.773. In *gamlss*, the intracluster correlation is defined as *ρ* = *σ*/(*σ* + 1), where *σ* = 1/(*α* + *β*) is the overdispersion parameter. The parameter *σ* has a log link function. Standard errors and confidence intervals for *σ* are obtained in the log scale. The relation between *ρ* and *σ* yields a confidence interval for *ρ*.

The estimate of the overall RR is highest (0.774) in the *bbmle* model, and its confidence interval is the longest (1.275). The estimate of the RR is lowest (0.645) in the conventional REM with ${\hat{\tau }}_{\text{REML}}^{2}=0.199$.

The results of this example can be compared with simulation results for *K* = 10 (in Figure 5), since it has nine studies. Thus, for ${\hat{\theta }}_{bbmle}=-0.257$, the bias of *bbmle* and *gamlss* is about 0.20, which leads to the estimate ${\hat{\theta }}_{\text{True}}=-0.257-0.20=-0.457$. For ${\hat{\theta }}_{\text{BD}}=-0.321$, the bias of the Breslow‐Day method for estimating *ρ* is 0.10, which leads to the estimate ${\hat{\theta }}_{\text{True}}=-0.321-0.10=-0.421$.

### 5.2 Example 2: Side effects of low‐dosage tricyclic antidepressants in acute‐phase depression

Systematic review^45^ in the Cochrane Library compared the effects and side effects of low‐dosage tricyclic antidepressants (TCA) with placebo and with standard‐dosage tricyclics in acute‐phase treatment of depression. Comparison 2 Outcome 6 is the meta‐analysis of the rate of side effects in the low‐dosage TCA group vs placebo. Table 3 gives the data on numbers of patients experiencing at least one side effect.

**Table 3:** Data on numbers of patients experiencing at least one side effect in studies of low‐dosage tricyclic antidepressants vs placebo. The study‐level estimate of log(RR), ${\hat{\theta }}_{j}$, comes from Equation 19

Study	*Y*_*jC*_	*n*_*jC*_	*Y*_*jC*_/*n*_*jC*_	*Y*_*jT*_	*n*_*jT*_	*Y*_*jT*_/*n*_*jT*_	${\hat{\theta }}_{j}$
1	17	28	0.607	16	24	0.667	0.092
2	7	10	0.700	12	12	1.000	0.336
3	3	12	0.250	8	13	0.615	0.810
4	30	62	0.484	29	60	0.483	−0.001
5	14	53	0.264	34	60	0.567	0.744
6	5	21	0.238	14	20	0.700	1.017
7	13	46	0.283	37	45	0.822	1.043
8	45	60	0.750	56	60	0.933	0.217
9	31	82	0.378	52	95	0.547	0.364
10	0	10	0.000	3	16	0.188	1.494
11	9	47	0.191	51	110	0.464	0.846
12	5	20	0.250	8	20	0.400	0.435
13	3	16	0.188	7	15	0.467	0.825
14	43	72	0.597	63	72	0.875	0.378
15	1	29	0.034	8	28	0.286	1.769
16	5	23	0.217	5	17	0.294	0.295

The incidence of side effects in the placebo group is substantial; only two of the 16 studies have *Y*_*jC*_/*n*_*jC*_ < 10*%*, and the highest is 75*%*. Again, the OR does not provide a satisfactory approximation for the RR. One would expect more patients in the treatment group to report side effects (*RR* > 1, *θ* > 0). The values of ${\hat{\theta }}_{j}$ show substantial heterogeneity, and they suggest a mixture of four groups: eight values from −0.001 to 0.435, six values from 0.744 to 1.043, one at 1.494, and one at 1.769.

Table 4 shows the estimated values of *τ* ^2^ in the conventional REM and of *ρ* in the BB model. The DerSimonian‐Laird estimate is ${\hat{\tau }}_{\text{DL}}^{2}=0.047$, and ${\hat{\tau }}_{\text{REML}}^{2}=0.068$. For the BB model, the six estimates of *ρ* again separate into two clumps: the method‐of‐moments, REML, MP, and BD estimates range from 0.006 to 0.031, and *bbmle* and *gamlss* both produce 0.175. From the simulation results in Figure 4 (especially those for *n* = 100 and *K* = 20), we might expect such a separation, but the values of the other parameters in the simulations are not close to the estimated values in this example.

**Table 4:** Point estimates and confidence intervals for τ ^2^, ρ, log‐risk‐ratio (LRR), and risk ratio (RR) in the example of side effects of low‐dosage tricyclic antidepressants vs placebo^a^

Model	Method	Overdispersion	L	U	LRR	L	U	Length	RR	L	U
		parameter						of CI			
		*τ* ^2^
FEM					0.355	0.258	0.452	0.194	1.426	1.294	1.571
REM	DL&IV	0.047	0.005	0.329	0.461	0.286	0.636	0.350	1.586	1.331	1.889
REM	REML&IV	0.068	0.005	0.275	0.480	0.286	0.674	0.388	1.616	1.331	1.961
		*ρ*
BB	MoM&IV	0.028	0.002	0.107	0.368	0.217	0.520	0.303	1.445	1.242	1.682
BB	REML&IV	0.031	0.004	0.109	0.369	0.214	0.525	0.311	1.447	1.238	1.690
BB	MP&IV	0.026	0.002	0.107	0.368	0.219	0.517	0.298	1.445	1.245	1.676
BB	BD&IV	0.006	−0.008	0.073	0.359	0.246	0.473	0.227	1.432	1.279	1.604
BB	bbmle	0.175	0.107	0.276	0.535	0.384	0.686	0.302	1.708	1.469	1.987
BB	gamlss	0.175	0.102	0.284	0.535	0.326	0.745	0.419	1.708	1.384	2.107

FEM is the fixed‐effect model, REM is the random‐effects model, and BB is the beta‐binomial model. *bbmle* and *gamlss* are beta‐binomial maximum‐likelihood‐based and generalized‐additive‐regression models. The heterogeneity parameter is *τ* ^2^ in REM and *ρ* in the BB model. *L* and *U* denote the lower and upper limits of the 95% confidence intervals (CIs).

Both *bbmle* and *gamlss* yield ${\hat{\pi }}_{T}=0.593$, ${\hat{\pi }}_{C}=0.347$, and hence *RR* = 1.708. This is likely to be a reasonable estimate of RR. From the simulation results in Figure 5, we would expect the other BB estimates to have negative bias. The estimates from the REM are also likely to be low. Of *bbmle* and *gamlss*, the latter has a substantially wider confidence interval.

## 6 RR IN COCHRANE REVIEWS

To explore the practical implications of the restricted range in meta‐analyses of RR, we reviewed random‐effects meta‐analyses that used RR in Cochrane Library Issue 4, 2004. As in the Cochrane Collaboration's Review Manager,^52^ we used inverse‐variance–weighted meta‐analysis and estimated the between‐study variance *τ* ^2^ by the DerSimonian‐Laird method. We also included the BB‐based analysis using *bbmle*.

We considered only the 2115 meta‐analyses with *K* ≥ 3 studies. Among those, 1286 MAs had ${\hat{\tau }}^{2}>0$ (by our calculations, using *metabin* from the R package *meta*). Those 1286 MAs included 8940 studies with *n*_*C*_ ≥ 5 and *n*_*T*_ ≥ 5.

For Study *j* in MA *m*, we calculated the estimated log(RR), ${\hat{\theta }}_{mj}$, from (19) and its within‐study variance *v*_*mj*_ from (21) with *ρ* = 0. (These calculations aim to minimize bias in ${\hat{\theta }}_{mj}$ and *v*_*mj*_. They add 1/2 to each cell of the 2 × 2 table for each study, whereas the conventional ones add 1/2 only when the 2 × 2 table contains a zero cell.) The FE weights are $w_{mj}^{F}=1/v_{mj}$, and the RE weights are $w_{mj}^{R}={\left(v_{mj}+{\hat{\tau }}_{m}^{2}\right)}^{-1}$. For the FEM and the REM, we set *w*_*mj*_ equal to $w_{mj}^{F}$ and $w_{mj}^{R}$, respectively, to obtain the combined effects ${\hat{\theta }}_{m}$ and their estimated variances $1/\sum_{j}w_{mj}$.

**Figure 7:**
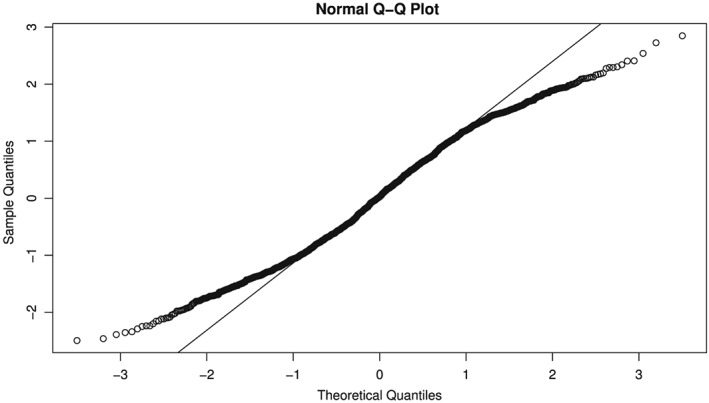
Normal Q‐Q plot of the studentized residuals for the studies from random‐effects model (REM) meta‐analyses of log‐risk‐ratio with $\hat{\theta }\geq 0$, ${\hat{\tau }}^{2}>0$ in Cochrane Library Issue 4

For the FEM and the REM, we calculated studentized residuals $r_{mj}=({\hat{\theta }}_{mj}-{\hat{\theta }}_{m})/s_{mj}$, now defining $s_{mj}^{2}=Var({\hat{\theta }}_{mj}-{\hat{\theta }}_{m})=1/w_{mj}-1/\sum_{j}w_{mj}$ for the respective weights. If, in the model for the log‐risk‐ratio, the assumption $b_{mj}∼N(0,\tau_{m}^{2})$ holds for these Cochrane reviews, these *r*_*mj*_ should have approximately the standard normal distribution. Because no single meta‐analysis involves enough studies to assess the reasonableness of that assumption, we combined the *r*_*mj*_ from all studies in MAs with $\hat{\theta }\geq 0$ and ${\hat{\tau }}^{2}>0$. The Q‐Q plot in Figure 7 shows that the distribution of the *r*_*mj*_ is not well approximated by a normal distribution. Although less striking, the corresponding plot of the *r*_*mj*_ from the studies in MAs with $\hat{\theta }<0$ and ${\hat{\tau }}^{2}>0$ (not shown) reinforces that message. The example in Section 5.2 suggests an additional departure: the studies' effects may come from a mixture of distributions. This could help to account for the appearance, in Figure 7, of a distribution whose tails are lighter than normal.

Building on the analysis in Section 3.2, we also inquire into the impact of the restriction in Equation 8. Because conventional meta‐analysis starts with the log of the sample RR, and the range of the log function is unbounded, it might seem that the restriction would have no impact. However, the basic data for each study include *y*_*jC*_ and *n*_*jC*_, and under the usual binomial model ${\hat{\pi }}_{jC}=y_{jC}/n_{jC}$ is an unbiased estimate of *π*_*jC*_. Thus, when ${\hat{\pi }}_{jT}=y_{jT}/n_{jT}>{\hat{\pi }}_{jC}$ and hence ${\hat{\theta }}_{j}>0$, larger *π*_*jC*_ (and hence $-\log\pi_{jC}$ closer to 0) will increase the impact of the restriction. We investigate the impact by estimating the probability of violating Equation 8, which we refer to as the truncation probability. For a normally distributed random effect *b*_*mj*_, this is approximated by $1-\Phi ((-\log({\hat{\pi }}_{mjC})-{\hat{\theta }}_{m})/{\hat{\tau }}_{m})$. We grouped these estimated probabilities into 10 bins: <0.05,[0.05 − 0.15),[0.15 − 0.25),…, ≥ 0.85. Table 5 shows the numbers of studies in each bin.

**Table 5:** Studies in 1286 meta‐analyses in the Cochrane Database that used REM for RR and had ${\hat{\tau }}^{2}>0$, cross‐classified by the estimated probability of truncation for π_T_ and whether $\hat{\theta }\geq 0$

	Estimated Probability of Truncation of *π*_*T*_				
	**<0.05**	**[0.05 − 0.15)**	**[0.15 − 0.25)**	**[0.25 − 0.35)**	**[0.35 − 0.45)**
$\hat{\theta }\geq 0$	1725	192	68	59	50
$\hat{\theta }<0$	6249	362	88	56	18
Total	7974	554	156	115	68
	**[0.45 − 0.55)**	**[0.55 − 0.65)**	**[0.65 − 0.75)**	**[0.75 − 0.85)**	**≥0.85**
$\hat{\theta }\geq 0$	16	22	16	14	5
$\hat{\theta }<0$	0	0	0	0	0
Total	16	22	16	14	5

**Figure 8:**
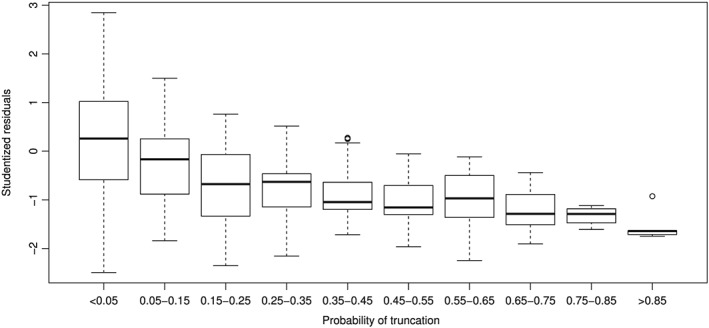
Boxplots of studentized residuals by truncation probability, for the studies from random‐effects model (REM) meta‐analyses of log‐risk‐ratio with $\hat{\theta }\geq 0$, ${\hat{\tau }}^{2}>0$ in Cochrane Library Issue 4

In total, 966 studies had truncation probability ≥0.05: 442 studies from 188 MAs with $\hat{\theta }\geq 0$ and 524 studies from 241 MAs with $\hat{\theta }<0$. These 429 MAs out of 1286 (exactly one‐third of the MAs using REM for RR) are likely to have reported biased conclusions. For the MAs with $\hat{\theta }\geq 0$ and ${\hat{\tau }}^{2}>0$, Figure 8 shows boxplots of the *r*_*mj*_ in each of the ten intervals of probability of truncation for *π*_*jT*_. The distance between ${\hat{\theta }}_{mj}$ and ${\hat{\theta }}_{m}$ is strongly related to the truncation probability. When the truncation probability is ≥0.35, the median of the *r*_*mj*_ is at or below −1. In these 123 studies, the violation of the assumptions of the conventional REM is likely to be greatest. Separate Q‐Q plots of the *r*_*mj*_ for the studies with truncation probability <0.35 and the studies with truncation probability ≥0.35 (not shown) support this conclusion.

**Figure 9:**
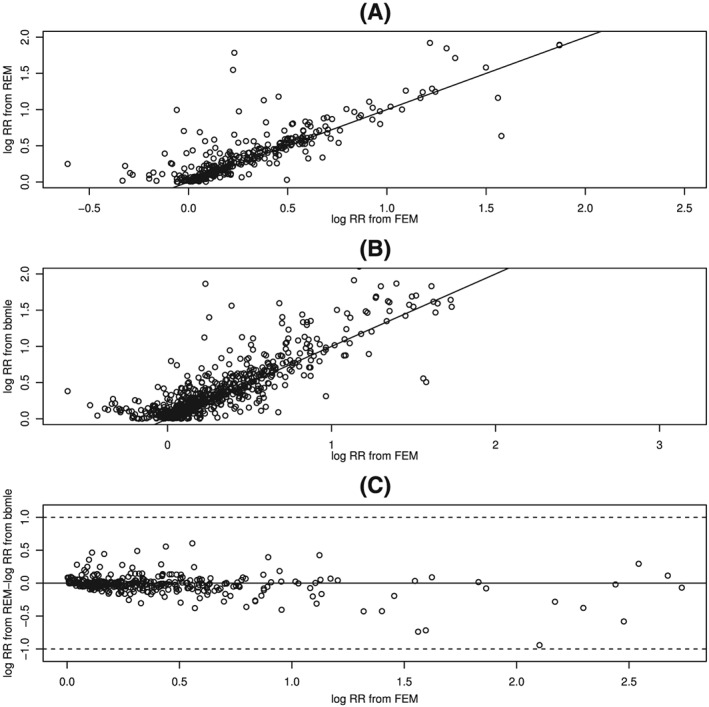
Scatterplot (vs log‐risk‐ratio from fixed‐effect model [FEM]) of the meta‐analytic estimates of log‐risk‐ratio obtained by: (A) random‐effects model (REM), for the 353 REM meta‐analyses of risk ratio (RR) with ${\hat{\theta }}_{\;\text{REM}}\geq 0$, ${\hat{\tau }}^{2}>0$; (B) bbmle, for the 713 meta‐analyses of RR with ${\hat{\theta }}_{bbmle}\geq 0$, $\hat{\rho }>0$; (C) difference between log(RR) from REM and bbmle for the 353 meta‐analyses with $\hat{\rho }>0$ and ${\hat{\theta }}_{bbmle}\geq 0$ and τ ^2^ > 0 and ${\hat{\theta }}_{\text{REM}}\geq 0$

The three panels of Figure 9 plot the estimates of log(RR) from the REM, the estimates of log(RR) from *bbmle*, and the difference between them versus the estimates from the FEM. For the 353 MAs with ${\hat{\theta }}_{\text{REM}}\geq 0$  and ${\hat{\tau }}^{2}>0$ in panel A, the majority of REM estimates are above their FEM counterparts, sometimes very substantially. This pattern supports the impression that the positive RRs and their significance reported from the conventional REM are overestimates. We conclude that the positive values of log(RR) estimated from REM are likely to be overestimates. Simulations performed previously to ascertain the quality of those estimates are likely to have provided downward‐biased results, compensating for this overestimation.

The *bbmle* estimates from the 713 MAs with ${\hat{\theta }}_{bbmle}\geq 0$ and $\hat{\rho }>0$ in panel B follow the same pattern, perhaps even more so. There are more positive values of $\hat{\theta }$ from *bbmle* than from REM, and they appear to have higher values. Relative to the estimates from the FEM, the differences between the estimates from the REM and those from *bbmle*, in panel C, are more often negative, and they are often not small. A difference of 0.223 in log(RR) corresponds to a factor of 1.25 in RR. These differences may be due to the differences in the underlying assumptions about the distribution of random effects in the two models, and hence to model misspecification in one of them, and/or to the biases discussed in Sections 3 and 4.

At the suggestion of a referee, we repeated the analyses of this section, restricting the participating meta‐analyses to only the 128 with *K* ≥ 10 studies, to guard against imprecise estimation of *τ* ^2^ for small *K*. The results, in Supporting Information (Figures S3‐S5), are qualitatively similar.

## 7 DISCUSSION

With models for log‐odds‐ratio as background, we have focused on models for meta‐analysis of log‐risk‐ratio, for two main reasons. First, when the event probabilities are not small, the RR is often more appropriate than the OR. Second, in generating binomial data for study‐level 2 × 2 tables under an REM for log‐risk‐ratio, one must impose a restriction to ensure that *π*_*jT*_ < 1 (in addition to having 0 < *π*_*jC*_ < 1). Thus, in the conventional REM, we explored the consequences of the restrictions on the parameter space. A small simulation study showed that they lead to bias in estimates of *τ* ^2^ and in the estimate of the overall log‐risk‐ratio.

The alternative of obtaining the data in the 2 × 2 tables from BB distributions, and using the log link function, has the same complications as in the log‐binomial model. In order to use the conventional meta‐analysis models for log(RR), one can estimate the *π*_*jT*_ and *π*_*jC*_ in each study by maximum likelihood or from the estimated logits in a BB regression with logit link. The variances of the resulting estimates of log(RR) involve *ρ* (intracluster correlation). We considered several ways of estimating *ρ*, but another small simulation study showed bias in estimates of *ρ* that was often unacceptable, though *bbmle* provided reasonable point and interval estimates of the overall log(RR) when the data were generated from BB distributions.

Thus, neither the log‐binomial model nor the BB model is satisfactory for meta‐analysis of (log of) RR. Because the range of the log function is unbounded, it might seem that conventional meta‐analysis of log(RR) would avoid the complications associated with restrictions on the parameter space, but it does not. Many meta‐analyses use (log of) RR as the effect measure (eg, in the Cochrane reviews summarized in Section 6), however, so reliable methods are needed.

Importantly, the standard log‐binomial‐normal model and the BB model are based on different assumptions about the unobservable mixing distributions. The results from these and similar two‐stage models are not always robust against violations of distributional assumptions. For instance, misspecification of the random‐effects distribution in GLMMs can induce bias in the estimates of the linear predictor parameters and severe bias in estimates of the variance components. Alonso et al 53 give a comprehensive discussion. For meta‐analysis of ORs using BB and REM, these misspecification biases are demonstrated in Bakbergenuly and Kulinskaya^33^ (Supplementary Material D). Unfortunately, it is very difficult to determine the true data‐generating mechanism for the random effects, especially when dealing with sparse data; Drikvandi et al^54^ discuss some developments.

For the log‐risk‐ratio, the complications in the log‐binomial model, Equation 6, arise from the restriction on *θ* introduced by the relation between *θ* and the nuisance parameters, $\alpha_{j}=\log(\pi_{jC})$. More technically, the joint range of *θ* and $\log(\pi_{C})$ is a proper subset of the set $\left\{(\log(\pi_{C}),\theta ):\log(\pi_{C})\leq 0,-\infty <\theta <+\infty \right\}$. By contrast, for the OR, −*∞* < logit(*π*_*C*_) < +*∞*, −∞<log(OR)<+∞, and the joint range of log(OR) and logit(*π*_*C*_) is the entire real plane. To circumvent this difficulty with the log‐risk‐ratio, Richardson et al^55^ propose a new nuisance parameter, the log of the odds‐product (OP). The log‐OP ranges from −*∞* to +*∞*, and this choice of nuisance parameter has the advantage that both log(RR) and the transformed risk difference, arctanh(RD)=log((1+RD)/(1−RD)), can be modeled independently of log(OP). The introduction of the log of the odds‐product as the nuisance parameter in models of log(RR) opens up a promising approach that should be the focus of substantial further research. However, it is plausible that this approach will suffer from transformation bias and other biases, which are the bane of the existing models for RR. In the interim, the use of OR instead of relative risk appears to be a safer option.

For the two examples in Section 5, the choice of method seems to matter. Although the confidence intervals overlap, the estimates of the overall log(RR) separate into three groupings: conventional REM with inverse‐variance weights, BB with inverse‐variance weights, and BB maximum likelihood. In interpreting the estimates, however, some caution is appropriate. The study‐level estimates of log(RR) in both examples suggest a mixture. Thus, a single distribution (as in the REMs) may not be an adequate description of the heterogeneity. Lin et al 56 argue that, if heterogeneity is present, it should permeate the entire collection of studies, instead of being limited to a small number of outlying studies. We add that presence of distinct groupings also represents a departure from regular heterogeneity. In such situations, it may be appropriate to model a cluster structure by a finite‐mixture distribution^57^ or a product‐partition model^58^ or to consider a fixed‐effects analysis.^59^

For a sizable number of meta‐analyses of RR in Cochrane reviews, we derived studentized residuals from the difference between the study‐level estimate and the overall estimate of the log‐risk‐ratio. When combined across meta‐analyses, the studentized residuals had a distribution that departed from a normal distribution, by having lighter tails. And when categorized into intervals of the estimated truncation probability, the studentized residuals showed a strong association between larger truncation probability and more‐negative difference between the study‐level estimate and the overall estimate. Because the vast majority of studentized residuals in meta‐analyses with $\hat{\theta }\geq 0$ belonged to the bins where the truncation probability was <0.35, we suspect upward bias in the overall estimates.

Other effect measures have restrictions on their parameter spaces. We would expect similar results for risk difference, response ratio, and arcsin( $\sqrt{p}$) for binomial proportions. The development of appropriate methodology for this important problem is an urgent task.

## CONFLICT OF INTEREST

The author reported no conflict of interest.

## Supporting information



Supporting info item
