# Supplementary material for: Pitfalls of using the risk ratio in meta‐analysis
Source: Res Synth Methods. 2019 Apr 11;10(3):398–419. doi: 10.1002/jrsm.1347 (PMC6767076; doi:10.1002/jrsm.1347)
Supplement: Supplementary file 1 — Supporting info item [file JRSM-10-398-s001.pdf]

Supplementary material

Appendix A

A.1 Estimation of ρ in the BB model

In this section, we give detailed derivations of the four estimators of ρ introduced in Section 4.5.

Method-of-moments estimator

Denote by $\sigma_j^2(\rho) = \mathrm{Var}(\hat{\theta}_j)$ the variance of log(RR) given in Equation (20). Then $\sigma_j^2(\rho) = \sigma_j^2(0) + a_j\rho$, where $\sigma_j^2(0)$ is the variance under the fixed-effect model and $a_j = (\pi_T^{-1} - 1)(1 - n_{jT}^{-1}) + (\pi_C^{-1} - 1)(1 - n_{jC}^{-1})$. The inverse-variance weights are $w_j(\rho) = \sigma_j^{-2}(\rho)$. Cochran's statistic is $Q = \sum \hat{w}_j(\hat{\theta}_j - \bar{\theta}_w)^2$, where $\hat{w}_j = \hat{\sigma}_j^{-2}(0)$ and $\bar{\theta}_w = \sum \hat{w}_j\hat{\theta}_j / \sum \hat{w}_j$. Under the null hypothesis of no over- or under-dispersion ($\rho = 0$), the Q-statistic has approximately a chi-squared distribution with $K - 1$ degrees of freedom, so $\mathrm{E}(Q) = K - 1$. Using the theoretical weights $w_j = w_j(0)$ instead of $\hat{w}_j$, the derivation of the expected value of Q yields

$$E(Q) = \sum w_j\sigma_j^2(\rho) - \frac{\sum w_j^2\sigma_j^2(\rho)}{\sum w_j}.$$

After substitution of $\sigma_j^2(\rho) = \sigma_j^2(0) + a_j\rho$, this becomes

$$E(Q) = K - 1 + \left[\sum w_j a_j - \frac{\sum w_j^2 a_j}{\sum w_j}\right]\rho.$$

The DerSimonian-Laird-inspired estimator of ρ, denoted by $\hat{\rho}_{MoM}$, is obtained by substituting Q for $E(Q)$ and the estimated weights $\hat{w}_j$ for the theoretical weights w_j and solving the resulting equation for ρ.

Modified Mandel-Paule estimator

Similarly, the modified Mandel-Paule estimator, $\hat{\rho}_{MP}$, is obtained by considering $Q(\rho) = \sum \hat{w}_j(\rho)(\hat{\theta}_j - \bar{\theta}_{w(\rho)})^2$ and solving the equation $Q(\rho) = K - 1$. The weighted mean is $\bar{\theta}_{w(\rho)} = \sum \hat{w}_j(\rho)\hat{\theta}_j / W(\rho)$, where $W(\rho) = \sum \hat{w}_j(\rho)$.

Restricted-maximum-likelihood estimator

Following Kulinskaya and Olkin[1], the REML equation for ρ is

$$(W(\rho))^{-1}\sum \hat{w}_j(\rho)\frac{a_j}{1 + a_j\rho} + \sum \hat{w}_j(\rho)(\theta_j - \theta)^2\frac{a_j}{1 + a_j\rho} = \sum \frac{a_j}{1 + a_j\rho}, \qquad \text{(A1)}$$

and an iterative procedure readily yields a solution, denoted by $\hat{\rho}_{REML}$.

Inverting the modified Breslow-Day test

The Breslow-Day test for homogeneity of odds ratios is based on the statistic

$$\sum_{j=1}^{K} \frac{(Y_{jT} - Y_{jT}(\hat{\psi}))^2}{\mathrm{Var}(Y_{jT}|\hat{\psi})},$$

where $Y_{jT}(\hat{\psi})$ and $\mathrm{Var}(Y_{jT}|\hat{\psi})$, respectively, denote the expected value and the asymptotic variance of the number of events in the treatment group of Study j based on the Mantel-Haenszel[2] (MH) estimate of the odds ratio, $\hat{\psi}$. We apply the same approach to the risk ratio.

The expected number of events, given RR $\hat{\psi}$ and the total number of events Y_j, is

$$Y_{jT}(\hat{\psi}) = \frac{\hat{\psi} n_{jT} Y_j}{n_j - n_{jT} + \hat{\psi} n_{jT}}.$$

The MH estimate for the relative risk is

$$\hat{\psi} = \frac{\sum y_{jT} n_{jC}/n_j}{\sum y_{jC} n_{jT}/n_j},$$

and the asymptotic variance[3] of Y_{jT} is

$$\begin{aligned} \mathrm{Var}(Y_{jT}|\hat{\psi}) = \; & \Big[\tfrac{1}{Y_{jT}(\hat{\psi})C_{jT}} + \tfrac{1}{(Y_j - Y_{jT}(\hat{\psi}))C_{jC}} + \\ & \tfrac{1}{(n_{jT} - Y_{jT}(\hat{\psi}))C_{jT}} + \tfrac{1}{(n_j - Y_j - n_{jT} + Y_{jT}(\hat{\psi}))C_{jC}}\Big]^{-1}, \end{aligned} \tag{A2}$$

where $C_{ji} = 1 + (n_{ji} - 1)\hat{\rho}$ are correction factors; $i = C, T$; $j = 1, \ldots, K$. The Breslow-Day statistic has a χ^2_{K-1} distribution, and this modification of it can be used for testing the homogeneity of RRs. Equating the BD statistic to $K - 1$ yields the Mandel-Paule-type estimate for ρ, denoted by $\hat{\rho}_{BD}$.

A.2 Function for meta-analysis of relative risk using bbmle

```
###########grp-level outcome data####################
###            |Event |No event|
### Treatment |ai     |bi      |
### Control   |ci     |di      |
### K is the number of studies
### ai and ci are number of events in T and C arms
###required packages : bbmle and emdbook
##initial parameters
my_bbmle<-function(ai,bi,ci,di) {
require(emdbook)
require(bbmle)
##initial parameters
pT0<-sum((ai+0.5)/(ni+1))/k
pC0<-sum((ci+0.5)/(ni+1))/k
rho0<-0.01
########################################
### negative log-likelihood functions for
### a pair of beta-binomial distribution
mtmp <- function(probT,sizeT,theta,probC,sizeC) {
-sum(dbetabinom(ai,probT,sizeT,theta,log=TRUE))
-sum(dbetabinom(ci,probC,sizeC,theta,log=TRUE))
}
m0 <- suppressWarnings(try(mle2(mtmp,start=list(probT=pT0,
theta=rho0^(-1)-1,probC=pC0),data=list(sizeT=ni,sizeC=ni)),
silent=TRUE))
if((!(inherits(m0, "try-error")))){
if((!(is.na(stdEr(m0)[[1]])))&(!(is.na(stdEr(m0)[[3]])))){
probT1<-coef(m0)[[1]]
probC1<-coef(m0)[[3]]
varT1<-(stdEr(m0)[[1]])^2
varC1<-(stdEr(m0)[[3]])^2
varlogRR<-(1/probT1)^2*varT1+(1/probC1)^2*varC1
RR1<-(probT1)/(probC1)
log_RR<-log(RR1)
logRRL<-log_RR-qnorm(.975)*sqrt(varlogRR)
logRRU<-log_RR+qnorm(.975)*sqrt(varlogRR)
rhoEst<-1/(coef(m0)[[2]]+1)
return(list(rhoEst,log_RR,logRRL,logRRU))
}}
}
```

A.3 Function for meta-analysis of relative risk using gamlss

```
##R version 3.5.0
############grp-level outcome data#######################
###              |Event |No event|
### Treatment |ai     |bi        |
### Control    |ci     |di        |
### K is the number of studies
### ai and ci are number of events in T and C arms
###required packages: gamlss
   my_gamlss<-function(ai,bi,ci,di) {
require(emdbook)
require(gamlss)
r<-c(ai,ci)
N<-c(ni,ni)
y<-cbind(r,N-r)
arm<-c(rep("T",times=k),rep("C",times=k))
arm<-as.factor(arm)
sink(tempfile())
modelGamlss <- suppressWarnings(try(gamlss(y~arm-1,sigma.
    formula=~1,family=BB), silent=TRUE))
sink()
if(!(inherits(modelGamlss, "try-error"))){
####fitting the model using gamlss##############
sink(tempfile())
res<-suppressWarnings(summary(modelGamlss,save=TRUE))
sink()
pC3<-exp(modelGamlss$mu.coefficients[[1]])/(1+exp(
    modelGamlss$mu.coefficients[[1]]))
pT3<-exp(modelGamlss$mu.coefficients[[2]])/(1+exp(
    modelGamlss$mu.coefficients[[2]]))
st.error1<-(res$coef.table[,2][[1]])
st.error2<-(res$coef.table[,2][[2]])
varC3<-(1/(1+exp(modelGamlss$mu.coefficients[[1]]))^2)^2*(st
    .error1)^2
varT3<-(1/(1+exp(modelGamlss$mu.coefficients[[2]]))^2)^2*(st
    .error2)^2
RR3<-pT3/pC3
logRRgamlss<-log(RR3)
varRR3<-varT3+varC3
logRRL_gamlss=0
logRRU_gamlss=0
```

```
if(!is.null(st.error2)){
logRRL_gamlss<-logRRgamlss-qnorm(.975)*sqrt(varRR3)
logRRU_gamlss<-logRRgamlss+qnorm(.975)*sqrt(varRR3)
}
rhoEst<-exp(modelGamlss$sigma.coefficients[[1]])/(1+exp(
    modelGamlss$sigma.coefficients[[1]]))
}
return(list(rhoEst,logRRgamlss,logRRL_gamlss,logRRU_gamlss))
}
```

A.4 Function for meta-analysis of relative risk using ρ_{DL}

```
##R version 3.5.0
###########grp-level outcome data####################
###            |Event |No event|
### Treatment |ai    |bi      |
### Control   |ci    |di      |
### K is the number of studies
### ai and ci are number of events in T and C arms
###required packages: None
my_DL_function_generic<-function(ai,ci,bi,di){
p1i<-(ai+0.5)/(ai+bi+1)
p2i<-(ci+0.5)/(ci+di+1)
psi_i<-(ai+0.5)*((ci+di)+0.5)/((ci+0.5)*(ai+bi+0.5))
yi      <- log(psi_i)
vi      <- 1/(ai+0.5)-1/(ai+bi+0.5)+1/(ci+0.5)-1/(ci+di+0.5)
n1i<-ai+bi
n2i<-ci+di
n_i<-n1i+n2i
Ri<-n1i/n2i
a_i<-(Ri*n_i/(Ri+1))*((1+psi_i-2*p1i)/(Ri*(1-p1i)+psi_i-p1i)
   )-1
wi<-1/vi
W<-sum(wi)
thetabar<-sum(yi*wi)/W
Q<-sum(wi*(yi-thetabar)^2)
barn_w<-sum(a_i*wi)/W
barn<-sum(a_i)/k
rho_M<-max((Q-k+1)/(k*barn-barn_w),-1/max(ni-1))
###estimated weights
w_M<-wi/(1+a_i*rho_M)
thetabar_M<-sum(yi*w_M)/sum(w_M)
Ltheta_M<-thetabar_M-qnorm(.975)/sqrt(sum(w_M))
Utheta_M<-thetabar_M+qnorm(.975)/sqrt(sum(w_M))
return(list(rho_M, thetabar_M, Ltheta_M, Utheta_M))
}
```

A.5 Function for meta-analysis of relative risk using ρ_{MP}

```
##R version 3.5.0
############grp-level outcome data######################
###              |Event |No event|
### Treatment |ai     |bi        |
### Control    |ci     |di        |
### K is the number of studies
### ai and ci are number of events in T and C arms
my_MP_function_generic<-function(ai,ci,bi,di){
k<-length(ai)
fMP<-function(g,sigma2,theta,a_i,k){
sum((theta-sum(theta/(sigma2*(1+a_i*g)))
/sum(1/(sigma2*(1+a_i*g))))^2/(sigma2*(1+a_i*g)))-(k-1)}
p1i<-(ai+0.5)/(ai+bi+1)
p2i<-(ci+0.5)/(ci+di+1)
psi_i<-(ai+0.5)*((ci+di)+0.5)/((ci+0.5)*(ai+bi+0.5))
yi     <- log(psi_i)
vi     <- 1/(ai+0.5) - 1/(ai+bi+0.5) + 1/(ci+0.5) - 1/(ci+di
   +0.5)
wi<-1/vi
n1i<-ai+bi
n2i<-ci+di
n_i<-n1i+n2i
Ri<-n1i/n2i
a_i<-(Ri*n_i/(Ri+1))*((1+psi_i-2*p1i)/(Ri*(1-p1i)+psi_i-p1i)
   )-1
ll<-max(-1/max(a_i),-1/max(n1i-1),-1/max(n2i-1))+0.00000001
uu<-1000
if (fMP(ll,sigma2=vi,theta=yi,a_i=a_i,k=k)*
fMP(uu,sigma2=vi,theta=yi,a_i=a_i,k=k)<0)
{
rho_MP<-as.numeric(uniroot(fMP, c(ll, uu), tol = 0.0001,
   sigma2=vi,theta=yi,a_i=a_i,k=k)[1])}
w_MP<-wi/(1+a_i*rho_MP)
thetabar_MP<-sum(yi*w_MP)/sum(w_MP)
if(rho_MP==(-1/max(ni-1))|is.na(thetabar_MP)|(rho_MP==(-1/
   max(ni-1))&is.na(thetabar_MP))){
yi_maxni<-yi[ni==max(ni)]
wi_maxni<-wi[ni==max(ni)]
thetabar_MP<-sum(yi_maxni*wi_maxni)/sum(wi_maxni)
w_MP<-wi[ni==max(ni)]
```

```
}
Ltheta_MP<-thetabar_MP-qnorm(.975)/sqrt(sum(w_MP))
Utheta_MP<-thetabar_MP+qnorm(.975)/sqrt(sum(w_MP))
return(list(rho_MP, thetabar_MP, Ltheta_MP, Utheta_MP))
}
```

A.6 Function for meta-analysis of relative risk using ρ_{BD}

```
##R version 3.5.0
############grp-level outcome data######################
###              |Event |No event|
### Treatment |ai     |bi       |
### Control   |ci     |di       |
### K is the number of studies
### ai and ci are number of events in T and C arms
###required packages: None
my_BD_function_generic<-function(ai,ci,bi,di){
nT<-ni
nC<-ni
XT<-ai
XC<-ci
k<-length(ai)
p1i<-(ai+0.5)/(ai+bi+1)
p2i<-(ci+0.5)/(ci+di+1)
psi_i<-(ai+0.5)*((ci+di)+0.5)/((ci+0.5)*(ai+bi+0.5))
yi<-log(psi_i)
vi<-1/(ai+0.5)-1/(ai+bi+0.5)+1/(ci+0.5)-1/(ci+di+0.5)
wi<-1/vi
n1i<-ai+bi
n2i<-ci+di
n_i<-n1i+n2i
Ri<-n1i/n2i
a_i<-(Ri*n_i/(Ri+1))*((1+psi_i-2*p1i)/(Ri*(1-p1i)+psi_i-p1i)
    )-1
#############################################
###########Breslow-day method##############
rho_BD<-numeric(1)
###Breslow-Day test conf. inter.
rho_BDU<-numeric(1)
rho_BDL<-numeric(1)
ll<-max(-1/max(a_i),-1/max(n1i-1),-1/max(n2i-1))+0.00000001
uu<-1000
##Mantel-Haenzsel estimator of relative risk
psiMH<-sum((nC*XT)/(nC+nT))/sum((nT*XC)/(nC+nT))
if(psiMH==1) {X1j=((ai+ci)*n1i)/(n1i+n2i)} else {
X1j<-psiMH*n1i*(ai+ci)/((n1i+n2i)-n1i+psiMH*n1i)
}
#function for the Standard Breslow day test
f<-function(rho,X_1j,X1j,xj,n1j,n2j,nj,k){
varj<-((1/(X1j*(1+(n1j-1)*rho))+1/((xj-X1j)*(1+(n2j-1)*rho))
```

```
+1/((n1j-X1j)*(1+(n1j-1)*rho))+1/((nj-xj-n1j+X1j)*(1+(n2j
-1)*rho)))^-1)
varj0<-((1/((X1j+.5)*(1+(n1j-1)*rho))+1/((xj-X1j+.5)*(1+(n2j
-1)*rho))+1/((n1j-X1j+.5)*(1+(n1j-1)*rho))+1/((nj-xj-n1j+
X1j+.5)*(1+(n2j-1)*rho)))^-1)
var<-ifelse(is.na(varj),varj0,varj)
var<-ifelse(var==0, varj0,var)
sum((X_1j-X1j)^2/var)-k+1
}

if(f(uu,ai,X1j,ai+ci,n1i,n2i,n1i+n2i,k=k)>0){rho_BD<-1}
if((f(ll,ai,X1j,ai+ci,n1i,n2i,n1i+n2i,k=k)>0)&(f(uu,ai,X1j,
ai+ci,n1i,n2i,n1i+n2i,k=k)<0)){
rho_BD<-as.numeric(uniroot(f,c(ll,uu),tol=0.00001,X_1j=ai,
X1j=X1j,xj=ai+ci,n1j=n1i,n2j=n2i,nj=(n1i+n2i),k=k)[1])
}
###Upper Limit
f1<-function(rho,X_1j,X1j,xj,n1j,n2j,nj,k){
varj<-((1/(X1j*(1+(n1j-1)*rho))+1/((xj-X1j)*(1+(n2j-1)*rho))
+1/((n1j-X1j)*(1+(n1j-1)*rho))+1/((nj-xj-n1j+X1j)*(1+(n2j
-1)*rho)))^-1)
varj0<-((1/((X1j+.5)*(1+(n1j-1)*rho))+1/((xj-X1j+.5)*(1+(n2j
-1)*rho))+1/((n1j-X1j+.5)*(1+(n1j-1)*rho))+1/((nj-xj-n1j+
X1j+.5)*(1+(n2j-1)*rho)))^-1)
var<-ifelse(is.na(varj),varj0,varj)
var<-ifelse(var==0, varj0,var)
sum((X_1j-X1j)^2/var)-qchisq(0.025,k-1)
}

if(f1(uu,ai,X1j,ai+ci,n1i,n2i,n1i+n2i,k=k)>0){rho_BDU<-1}
if((f1(ll,ai,X1j,ai+ci,n1i,n2i,n1i+n2i,k=k)>0)&(f1(uu,ai,X1j
,ai+ci,n1i,n2i,n1i+n2i,k=k)<0)){
rho_BDU<-as.numeric(uniroot(f1,c(ll,uu),tol=0.00001,X_1j=ai,
X1j=X1j,xj=ai+ci,n1j=n1i,n2j=n2i,nj=(n1i+n2i),k=k)[1])
}
##Lower Limit
f2<-function(rho,X_1j,X1j,xj,n1j,n2j,nj,k){
varj<-((1/(X1j*(1+(n1j-1)*rho))+1/((xj-X1j)*(1+(n2j-1)*rho))
+1/((n1j-X1j)*(1+(n1j-1)*rho))+1/((nj-xj-n1j+X1j)*(1+(n2j
-1)*rho)))^-1)
varj0<-((1/((X1j+.5)*(1+(n1j-1)*rho))+1/((xj-X1j+.5)*(1+(n2j
-1)*rho))+1/((n1j-X1j+.5)*(1+(n1j-1)*rho))+1/((nj-xj-n1j+
X1j+.5)*(1+(n2j-1)*rho)))^-1)
var<-ifelse(is.na(varj),varj0,varj)
```

```
var<-ifelse(var==0, varj0,var)
sum((X_1j-X1j)^2/var)-qchisq(0.975,k-1)
}

if(f2(uu,ai,X1j,ai+ci,n1i,n2i,n1i+n2i,k=k)>0){rho_BDL<-ll}
if((f2(ll,ai,X1j,ai+ci,n1i,n2i,n1i+n2i,k=k)>0)&(f2(uu,ai,X1j
    ,ai+ci,n1i,n2i,n1i+n2i,k=k)<0)){
rho_BDL<-as.numeric(uniroot(f2,c(ll,uu),tol=0.00001,X_1j=ai,
    X1j=X1j,xj=ai+ci,n1j=n1i,n2j=n2i,nj=(n1i+n2i),k=k)[1])
}
####Inverse variance estimation of theta using rho_BD
    ##################
w_BD<-wi/(1+a_i*rho_BD)
thetabar_BD<-sum(yi*w_BD)/sum(w_BD)
Ltheta_BD<-thetabar_BD-qnorm(.975)/sqrt(sum(w_BD))
Utheta_BD<-thetabar_BD+qnorm(.975)/sqrt(sum(w_BD))
return(list(rho_BD,rho_BDL,rho_BDU,thetabar_BD,Ltheta_BD,
    Utheta_BD))

}
```

Appendix B

B.1 Estimation of the between-studies variance when $\tau^2 = 0.1$ and the data are generated under the point-mass option and the truncation option

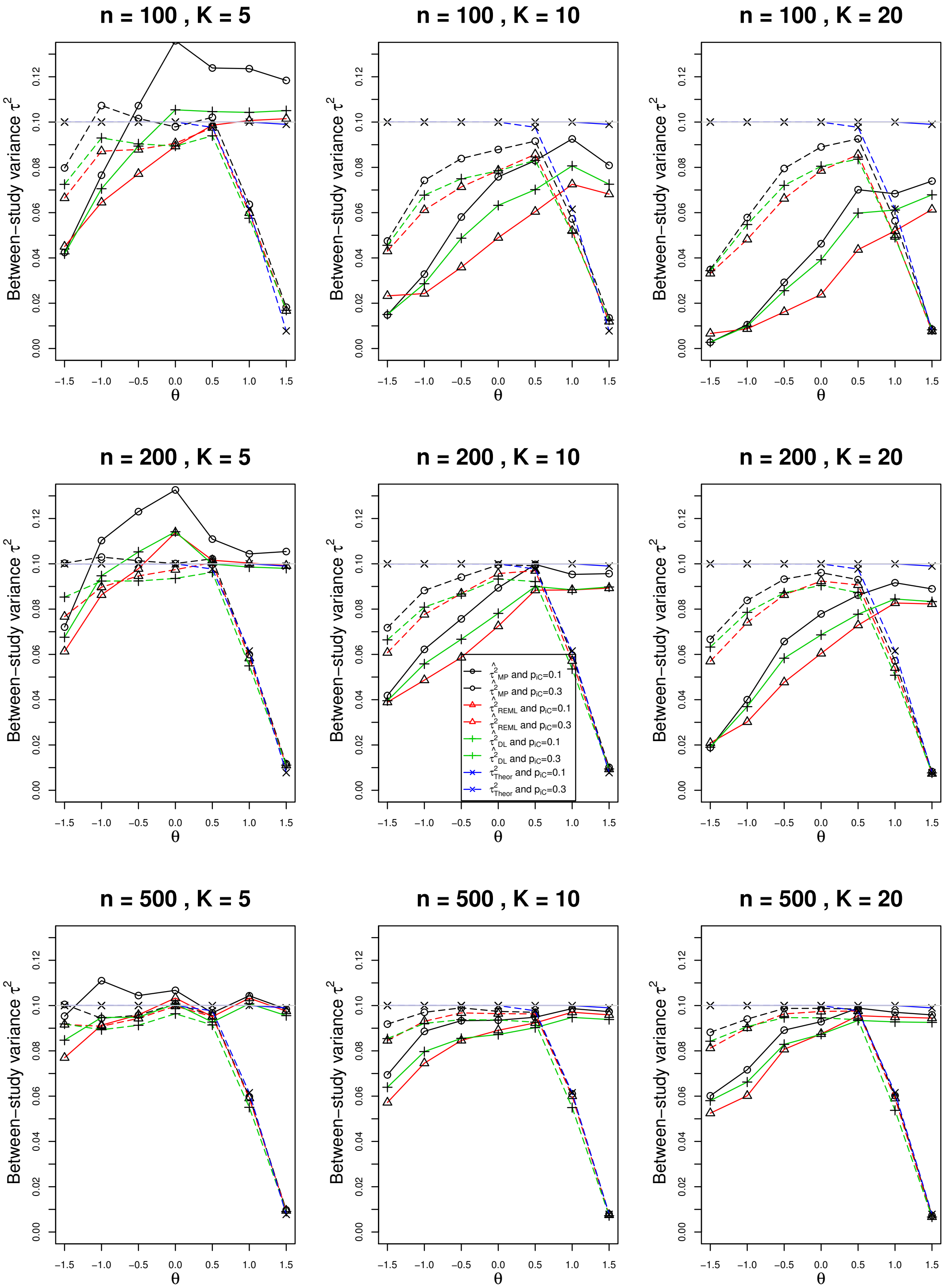

Figure B.1: Relation of estimates of the between-studies variance τ^2 to the overall log-risk-ratio (θ) in K studies, each of total sample size n, when data come from the binomial-normal model with point mass for $\tau^2 = 0.1$ and $\pi_{jC} = 0.1$ (solid lines) and 0.3 (dashed). The Mandel-Paule (circle), REML (triangle), and DerSimonian-Laird (plus) estimation methods are compared with the true variance (cross). Light grey line at 0.1.

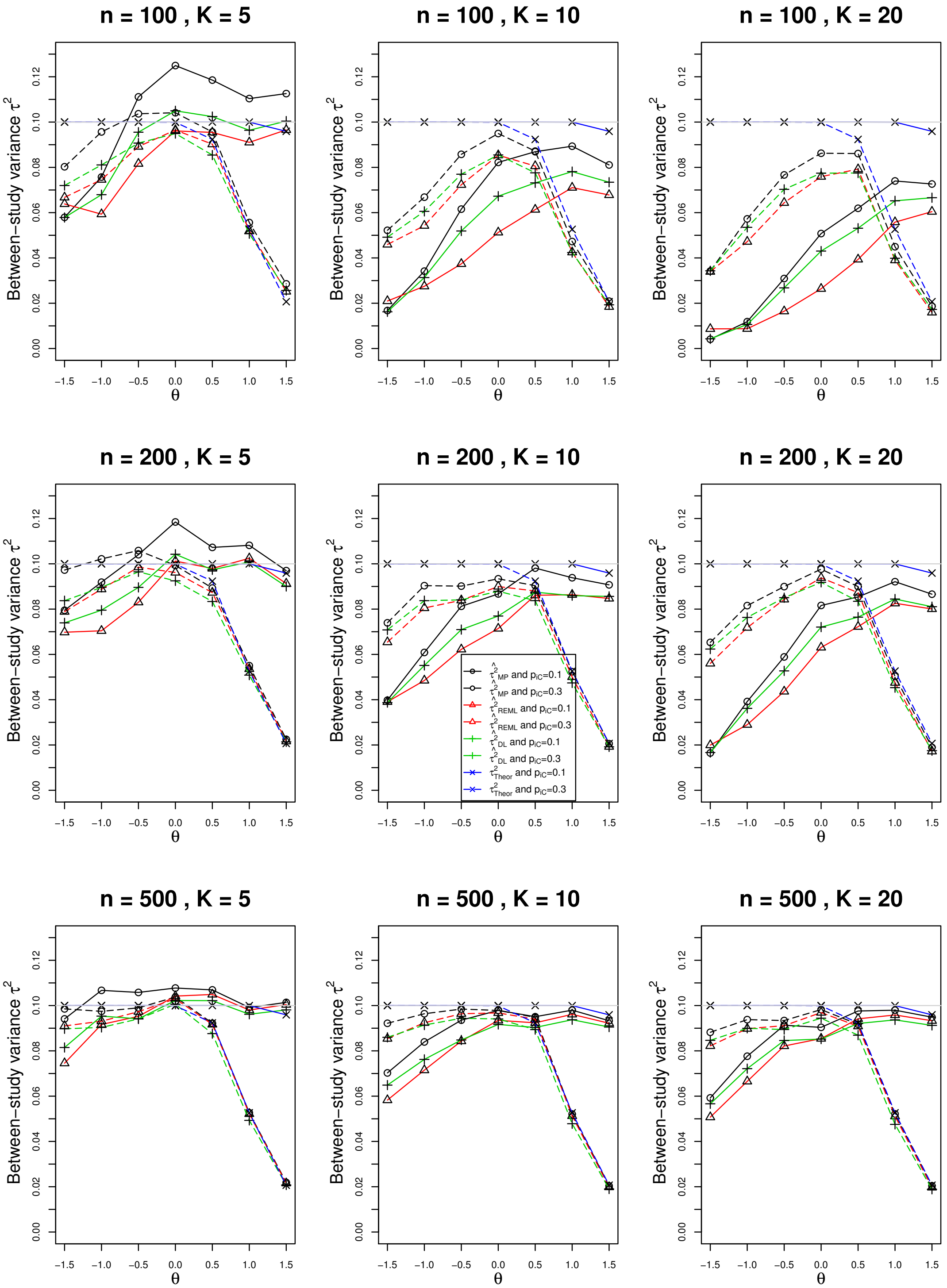

Figure B.2: Relation of estimates of the between-studies variance τ^2 to the overall log-risk-ratio (θ) in K studies, each of total sample size n, when data come from the binomial-normal model with truncation for $\tau^2 = 0.1$ and $\pi_{jC} = 0.1$ (solid lines) and 0.3 (dashed). The Mandel-Paule (circle), REML (triangle), and DerSimonian-Laird (plus) estimation methods are compared with the true variance (cross). Light grey line at 0.1.

B.2 Figures for studentized residuals when $K \geq 10$

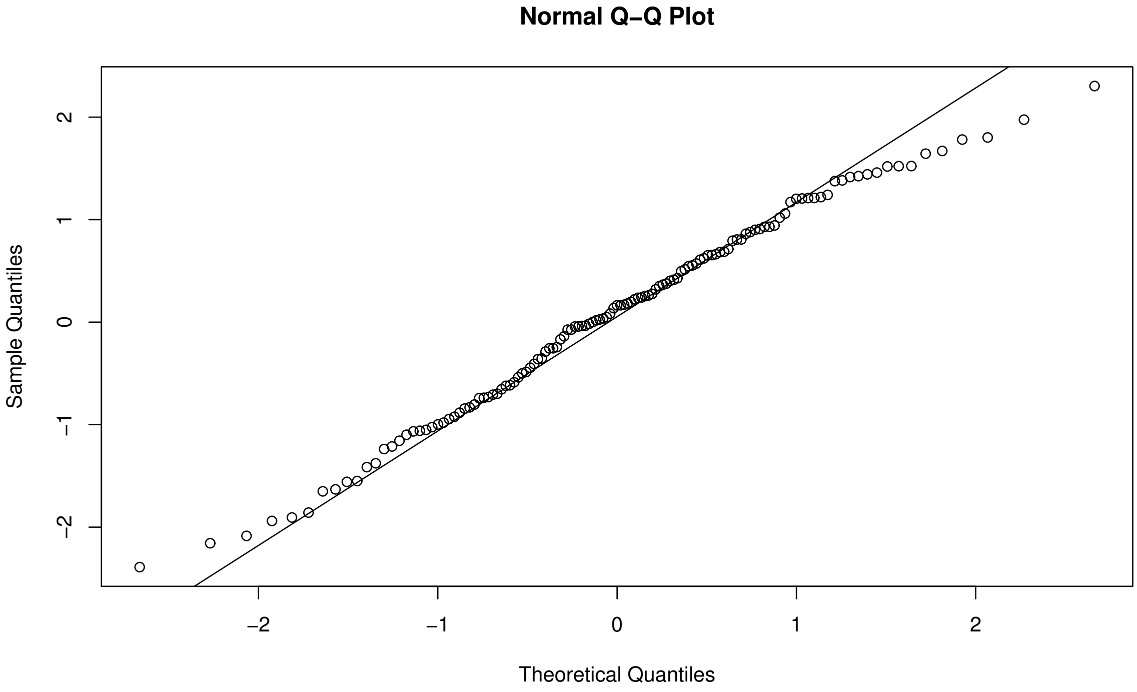

,

Figure B.3: Normal Q-Q plot of the 129 studentized residuals for the studies from 11 REM meta-analyses of RR with $\hat{\theta} \geq 0$, $\hat{\tau}^2 > 0$ and $K \geq 10$ in Cochrane Library Issue 4.

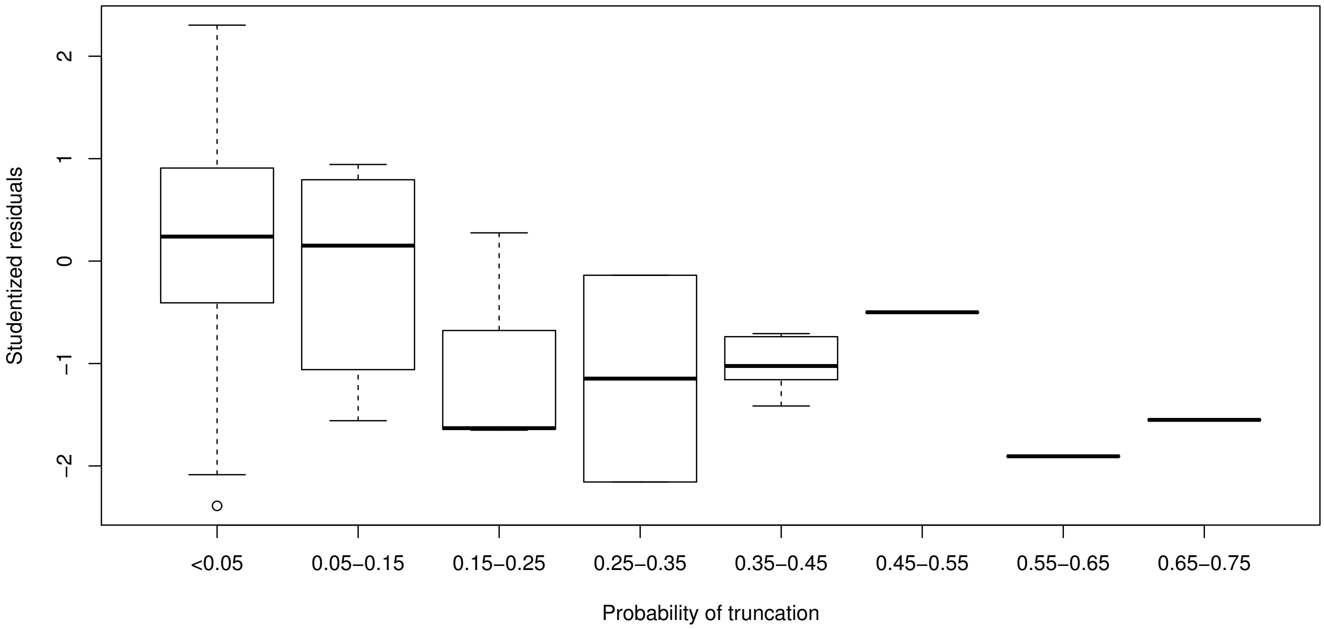

Figure B.4: Boxplots of 129 studentized residuals by truncation probability, for the studies from 11 REM meta-analyses of RR with $\hat{\theta} \geq 0$, $\hat{\tau}^2 > 0$ and $K \geq 10$ in Cochrane Library Issue 4.

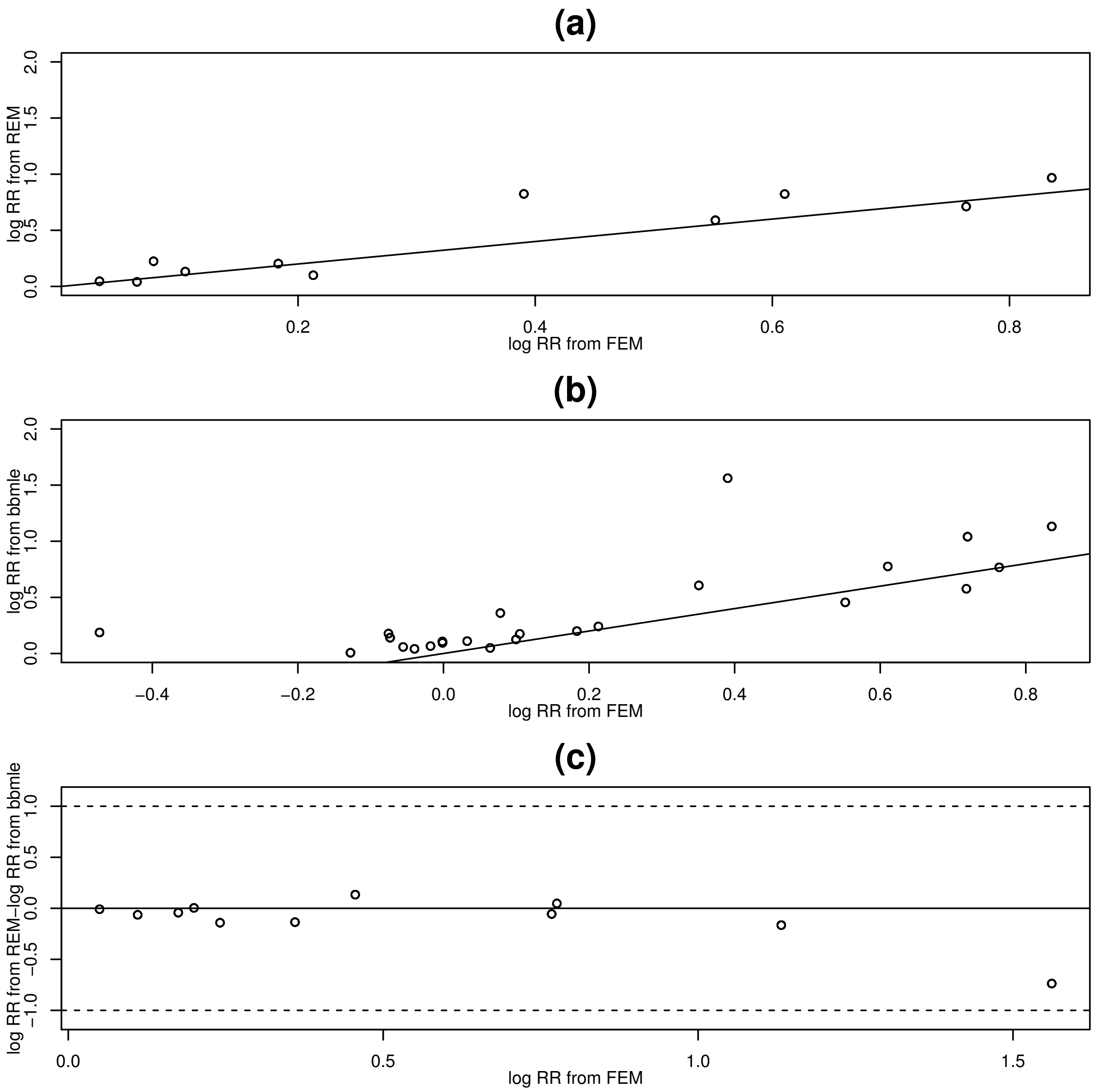

Figure B.5: Scatterplot (vs. log-risk-ratio from FEM) of the meta-analytic estimates of log-risk-ratio obtained by:(a) REM, for the 11 REM meta-analyses of RR with $K \geq 10$, $\hat{\theta}_{REM} \geq 0$ and $\hat{\tau}^2 > 0$; (b) *bbmle*, for the 24 meta-analyses of RR with $K \geq 10$, $\hat{\theta}_{bbmle} \geq 0$ and $\hat{\rho} > 0$; (c) Difference between log(RR) from REM and *bbmle* for the 11 meta-analyses with $K \geq 10$, $\hat{\rho} > 0$ and $\hat{\theta}_{bbmle} \geq 0$ and $\tau^2 > 0$ and $\hat{\theta}_{REM} \geq 0$.

References

[1] E. Kulinskaya and I. Olkin, "An overdispersion model in meta-analysis," *Statistical Modelling*, vol. 14, no. 1, pp. 49–76, 2014.

[2] N. Mantel and W. Haenszel, "Statistical aspects of the analysis of data from retrospective studies of disease," *Journal of the National Cancer Institute*, vol. 22, no. 4, pp. 719–748, 1959.

[3] G. A. Darlington and A. Donner, "Meta-analysis of community-based cluster randomization trials with binary outcomes," *Clinical Trials*, vol. 4, no. 5, pp. 491–498, 2007.
